# Autophagy in *Pseudomonas aeruginosa* infection: host defense, bacterial countermeasures, and therapeutic targeting

**DOI:** 10.3389/fmicb.2026.1976209

**Published:** 2026-10-12

**Authors:** Bingbing Zhang, Yanling Huang, Lingling Wen, Qiaoli Hua, Lijun Zhao, Jiangang Shen, Meiling Zhu

**Affiliations:** 1 The Fourth Clinical Medical College of Guangzhou University of Chinese Medicine, Shenzhen, China; 2 Shenzhen Traditional Chinese Medicine Hospital, Shenzhen, China; 3 Shenzhen Hospital of Integrated Traditional Chinese and Western Medicine, Shenzhen, China; 4 School of Chinese Medicine, The University of Hong Kong, Pok Fu Lam, Hong Kong SAR, China

**Keywords:** autophagy, bacterial persistence, inflammasome, lysosome, mitophagy, *Pseudomonas aeruginosa*, xenophagy

## Abstract

*Pseudomonas aeruginosa* (*P. aeruginosa*) is a major opportunistic pathogen whose diverse virulence strategies, increasing antibiotic resistance, and ability to establish persistent infections continue to compromise the efficacy of conventional antimicrobial therapies. Although traditionally regarded as an extracellular pathogen, *P. aeruginosa* can also enter and persist within host cells, triggering inflammatory responses and cellular injury while simultaneously modulating host autophagic responses. On the one hand, xenophagy and related autophagic processes recognize and deliver intracellular bacteria for lysosomal degradation. Mitophagy eliminates damaged mitochondria generated during infection and limits the accumulation of mitochondrial danger signals. Meanwhile, autophagy regulates inflammasome activation by controlling the removal of damaged organelles and inflammasome components, thereby shaping inflammatory responses during infection. Collectively, these processes contribute to the protective role of autophagy in host defense against *P. aeruginosa* infection. On the other hand, *P. aeruginosa* can interfere with or remodel host autophagy through secreted effector molecules, quorum sensing signals, and infection-induced host signaling pathways, resulting in a functional disconnect between autophagic activation and effective bacterial clearance or other antimicrobial responses. This review summarizes current advances in understanding the interplay between autophagy and *P. aeruginosa*, highlighting the protective functions of autophagy in host defense, the countermeasures employed by *P. aeruginosa* to evade autophagic clearance, and the therapeutic implications of these host–pathogen interactions. These insights may support the development of host-directed therapeutic approaches targeting autophagy to improve antibacterial defense against *P. aeruginosa*.

## 1 Introduction

*Pseudomonas aeruginosa* (*P. aeruginosa*) is a prominent opportunistic pathogen and a major cause of antimicrobial-resistant infections worldwide. In the Global Burden of Disease 2019 assessment of bacterial antimicrobial resistance, *P. aeruginosa* was among the six pathogens responsible for the greatest number of deaths attributable to antimicrobial resistance. When infections caused by both susceptible and resistant strains are considered, *P. aeruginosa* infections were associated with approximately 559,000 deaths and 18.9 million years of life lost worldwide (Antimicrobial Resistance Collaborators, 2022). Carbapenem resistance markedly exacerbates this burden. In bloodstream infections caused by *P. aeruginosa*, carbapenem resistance is an independent predictor of mortality, with a pooled attributable mortality of approximately 8–18% and an adjusted odds ratio of approximately 3 (Zhang et al., 2016). Given the substantial clinical burden and limited therapeutic options, carbapenem-resistant *P. aeruginosa* is classified as a high-priority pathogen in the 2024 World Health Organization Bacterial Priority Pathogens List (World Health Organization, 2024).

Acute *P. aeruginosa* pulmonary infection is commonly characterized by rapid epithelial barrier damage, neutrophil recruitment, and increased expression of virulence factors, whereas chronic airway infection more often involves progressive bacterial adaptation, including biofilm formation, altered quorum sensing regulation, and sustained exposure of host cells to secreted bacterial effectors (Sadikot et al., 2005; Parkins et al., 2018; Folkesson et al., 2012). In intensive care units, *P. aeruginosa* is one of the most common pathogens causing ventilator-associated pneumonia (Ramírez-Estrada et al., 2016). In patients with cystic fibrosis (CF), persistent airway colonization by *P. aeruginosa* represents a major driver of disease progression and premature mortality (Parkins et al., 2018). The difficulty in eliminating established *P. aeruginosa* infections is associated not only with the production of diverse virulence factors but also with antimicrobial resistance mechanisms and chronic persistence (Hauser, 2009; Lister et al., 2009; Pang et al., 2019). These features substantially reduce the effectiveness of conventional antimicrobial therapies, while the development of new antibiotics has not fully kept pace with clinical needs (Tacconelli et al., 2018). Therefore, research on *P. aeruginosa* infections has increasingly shifted from directly targeting the pathogen toward host-directed therapeutic strategies. This approach aims to enhance endogenous antimicrobial capacity or restore impaired cellular functions that contribute to pathogen persistence, thereby promoting bacterial clearance, reducing the chronic persistence of *P. aeruginosa* within the host, and limiting excessive inflammatory damage caused by infection (Kaufmann et al., 2018; Maurice et al., 2018).

Autophagy is a lysosome-dependent intracellular degradation system that maintains cellular homeostasis by enclosing damaged organelles, protein aggregates, and invading microorganisms within autophagosomes, which subsequently fuse with lysosomes for cargo degradation and recycling (Mizushima and Komatsu, 2011; Levine and Kroemer, 2019). Beyond its fundamental role in cellular quality control and organelle surveillance, autophagy also contributes to antimicrobial defense and the regulation of inflammatory responses during infection (Gomes and Dikic, 2014; Deretic, 2021). Therefore, autophagy has emerged as a potential target for therapeutic approaches against infections caused by pathogens such as *P. aeruginosa*.

However, increasing evidence indicates that the interaction between pathogens and autophagy does not necessarily lead to effective antimicrobial protection. Under the selective pressure imposed by microbial restriction mediated by host autophagy and cellular damage control, *P. aeruginosa* has evolved diverse strategies to inhibit, redirect, or exploit autophagic processes, thereby promoting bacterial survival and persistence (Jabir et al., 2015; Li et al., 2015; Jiang et al., 2016). Thus, autophagy represents a complex process during *P. aeruginosa* infection, with outcomes influenced by the balance between effective antimicrobial defense and pathogen-mediated interference. A comprehensive understanding of this bidirectional interaction and the mechanisms that determine its outcome will be essential for developing strategies that modulate autophagy to improve defense against *P. aeruginosa* infection.

This review discusses the interplay between autophagy and *P. aeruginosa* from the perspectives of host defense, bacterial countermeasures, and therapeutic modulation. We focus on infection-related autophagy subtypes, including xenophagy and mitophagy, as well as their interaction networks with inflammasomes. Furthermore, we summarize the strategies employed by *P. aeruginosa* to suppress or hijack autophagic responses and evaluate the therapeutic potential of modulating autophagy toward protective outcomes.

## 2 Infection-related autophagy

Autophagy broadly comprises macroautophagy, microautophagy, and chaperone-mediated autophagy (CMA) (Galluzzi et al., 2017; Yim and Mizushima, 2020). Macroautophagy involves the formation of double-membrane autophagosomes that deliver cytoplasmic cargo to lysosomes, whereas microautophagy directly incorporates cytoplasmic material through lysosomal or late-endosomal membrane remodeling (Mizushima and Levine, 2010; Oku and Sakai, 2018). CMA selectively translocates KFERQ-like motif-containing soluble proteins across the lysosomal membrane through heat shock cognate 70 (HSC70) and lysosome-associated membrane protein 2A (LAMP2A) (Kaushik and Cuervo, 2018). Among these pathways, macroautophagy has been most extensively implicated in host responses to *P. aeruginosa* infection and therefore constitutes the major focus of this review.

Following recognition of invading bacteria, infection-associated damaged mitochondria, or inflammatory signals, macroautophagy initiates the formation of isolation membranes that expand to engulf cytoplasmic components, generating double membrane-bound autophagosomes. These autophagosomes subsequently fuse with late endosomes or lysosomes, allowing the enclosed cargo to be degraded by lysosomal acidic hydrolases.

Through selective recognition and processing of these substrates, autophagic responses influence bacterial elimination, cellular stress responses, and inflammatory regulation. Moreover, the outcome of autophagy during *P. aeruginosa* infection is influenced by bacterial characteristics.

### 2.1 Recognition of *Pseudomonas aeruginosa* and induction of autophagy

Autophagy can be rapidly activated during *P. aeruginosa* infection in response to bacterial sensing and cellular stress. Pattern-recognition receptors provide an important link between bacterial sensing and the autophagy machinery. In macrophages, *P. aeruginosa* induces autophagy through Toll-like receptor 4 (TLR4) and its adaptor TIR-domain-containing adapter-inducing interferon-β (TRIF), whereas activation of the NLRC4–caspase-1 pathway can counteract this response by cleaving TRIF (Jabir et al., 2015). Toll-like receptor 2 (TLR2)-dependent activation of the Src-family kinase Lyn has also been implicated in the recruitment of autophagy machinery to bacteria-containing compartments in alveolar macrophages (Li et al., 2016). NOD-like receptor signaling may further modulate this response, although its effects are not uniform; for example, NLRP3-associated signaling has been reported to influence autophagic responses in human macrophages, whereas NLRC4 activation can restrict TLR4–TRIF-dependent autophagy (Deng et al., 2016; Jabir et al., 2015).

In addition to receptor-mediated signaling, cellular stress caused by infection can activate several upstream regulators of autophagy. In PAO1-infected alveolar macrophages, Annexin A2-associated signaling reduces Akt–mTOR activity and promotes ULK1/2-dependent autophagosome formation (Li et al., 2015). Bacterial virulence factors can also serve as potent stress signals. Pyocyanin produced by *P. aeruginosa* induces autophagy through the GCN2–eIF2α–ATF4 integrated stress response, whereas the H2 type VI secretion system (H2-T6SS) effector TplE triggers endoplasmic reticulum stress and activates the IRE1α–XBP1 pathway, leading to increased autophagic activity (Yang et al., 2016; Jiang et al., 2016). Mitochondrial dysfunction and oxidative stress caused by infection provide additional signals that are closely linked to autophagic and mitophagic responses.

Thus, *P. aeruginosa*-induced autophagy arises from the convergence of innate immune recognition, stress-sensitive signaling, and organelle damage. Importantly, induction of autophagy represents only the initial response to infection and does not necessarily indicate successful completion of the degradative pathway. Whether these signals ultimately result in effective autophagic flux depends on the subsequent formation, maturation, and lysosomal degradation of autophagic cargo, as discussed below.

### 2.2 Canonical autophagy machinery and autophagic flux

Although multiple signaling pathways can initiate autophagy during *P. aeruginosa* infection, successful progression through the autophagy–lysosome pathway requires the coordinated operation of the core autophagy machinery and the establishment of functional autophagic flux (Levine and Kroemer, 2019).

The ULK1/2 complex acts as a central regulator of autophagy initiation and responds to changes in nutrient availability, cellular stress, and growth factor signaling (Hosokawa et al., 2009). Under nutrient-rich conditions, mechanistic target of rapamycin complex 1 (mTORC1) suppresses ULK1/2 activity, whereas inhibition of mTORC1 or activation of stress-responsive pathways promotes ULK complex activation and initiates autophagosome formation (Kim et al., 2011). Activated ULK1/2 subsequently regulates the Beclin-1–VPS34 class III phosphatidylinositol 3-kinase complex, resulting in the generation of phosphatidylinositol-3-phosphate (PI3P)-enriched membrane domains that serve as platforms for autophagosome nucleation (Russell et al., 2013).

The elongation and closure of autophagosomal membranes depend on two ubiquitin-like conjugation systems. The ATG12–ATG5–ATG16L1 complex facilitates the recruitment of LC3, which is conjugated with phosphatidylethanolamine to form LC3-II and become incorporated into the autophagosomal membrane (Hanada et al., 2007; Kabeya et al., 2000; Mizushima et al., 2003). Following cargo sequestration, mature autophagosomes undergo trafficking and fusion with lysosomes through Rab7-dependent transport and SNARE/HOPS-mediated membrane fusion, ultimately forming autolysosomes where enclosed materials are degraded and recycled (Gutierrez et al., 2004b; Jiang et al., 2014).

Assessment of autophagy activity cannot rely on changes in individual markers alone. Therefore, autophagic flux provides a more accurate indication of whether cargo is successfully transported to and degraded within lysosomes (Klionsky et al., 2021). During *P. aeruginosa* infection, this distinction is particularly important because bacterial factors can affect different stages of the autophagy pathway (Heimer et al., 2013; Rao et al., 2021). Enhanced initiation of autophagy does not necessarily lead to effective degradation of bacterial cargo if subsequent autophagosome maturation, lysosomal acidification, or cargo degradation is impaired. Understanding this distinction is essential for interpreting the diverse roles of autophagy during *P. aeruginosa* infection, where both host-protective functions and bacterial interference with the pathway will be discussed below.

### 2.3 Strain-specific regulation of autophagy by *Pseudomonas aeruginosa*

Although *P. aeruginosa* is closely associated with autophagy regulation, most mechanistic studies investigating *P. aeruginosa*-induced autophagy have relied on laboratory strains, particularly PAO1, PA14, and PAK. Given the limited evidence currently available, the specific relationship between *P. aeruginosa* and autophagy may also be related to bacterial genetic background, virulence factors, and adaptation during infection.

PAO1 has been widely used as the primary model for elucidating the protective role of autophagy during *P. aeruginosa* infection. Studies using PAO1 have demonstrated that infection induces ATG-dependent autophagic responses in macrophages and epithelial cells, whereas disruption of core autophagy genes impairs the control of intracellular bacteria (Yuan et al., 2012; Li et al., 2015). These findings established the initial concept that autophagy serves as an antimicrobial defense mechanism against *P. aeruginosa*. However, subsequent studies have revealed that bacterial factors can actively reshape this response.

PA14 represents another widely studied strain with distinct virulence characteristics. Compared with PAO1, PA14 produces higher levels of phenazines and exhibits stronger expression of several virulence-associated traits. Pyocyanin, a redox-active phenazine produced by PA14, induces autophagy in airway epithelial cells through the GCN2–eIF2α–ATF4 stress response pathway (Yang et al., 2016). This observation highlights that bacterial metabolites, rather than only direct bacterial invasion, can influence autophagy activation.

PAK is a commonly used strain carrying an active type III secretion system (T3SS), which represents another important factor influencing autophagic responses. ExoS, a T3SS effector protein frequently associated with PAK, inhibits VPS34-dependent phosphatidylinositol-3-phosphate production, thereby reducing autophagosome formation (Rao et al., 2021). In addition, PAK infection has been shown to impair lysosomal function through inhibition of TFEB activation mediated by host calpain-1, resulting in defective autophagic degradation (Wu et al., 2025). These findings indicate that a specific bacterial strain may affect multiple stages of the autophagy pathway, including autophagosome formation and completion of autophagic flux.

Strains producing different T3SS effectors exhibit distinct effects on host responses. ExoU-producing strains, such as PA103, display highly cytotoxic phenotypes characterized by membrane damage, mitochondrial dysfunction, and inflammatory responses. Although direct evidence linking PA103 to specific autophagy pathways remains limited, ExoU-mediated cellular injury may influence mitochondrial quality control and autophagic responses (Sato et al., 2003). In contrast, ExoS-producing strains are more frequently associated with chronic respiratory infections and can interfere with host intracellular trafficking and antimicrobial processes through their ADP-ribosyltransferase activity (Hauser, 2009; Barbieri and Sun, 2004). Thus, differences in T3SS effector profiles among *P. aeruginosa* strains may contribute to variations in host autophagic responses.

Clinical isolates provide additional evidence that autophagic responses induced by *P. aeruginosa* are not restricted to laboratory strains. In a study using nine keratitis-associated clinical isolates, PAO1, and T3SS-deficient mutants, all clinical isolates were able to induce autophagy in human corneal epithelial cells, whereas T3SS-deficient strains showed reduced autophagy induction and altered intracellular survival characteristics. Pharmacological activation of autophagy further enhanced bacterial clearance for isolates associated with poorer clinical outcomes, suggesting that strain-specific virulence profiles, particularly T3SS activity, can influence the interaction between *P. aeruginosa* and host autophagy (Mohankumar et al., 2018).

Long-term adaptation during chronic infection introduces another layer of complexity into the interaction between *P. aeruginosa* and host autophagy. In the airways of patients with cystic fibrosis (CF), *P. aeruginosa* undergoes extensive evolutionary changes, including enhanced biofilm formation, altered quorum sensing regulation, reduced expression of acute virulence factors, and metabolic adaptation (Smith et al., 2006). Although direct comparisons of autophagic flux between CF-adapted isolates and acute infection strains remain limited, studies in CF models have demonstrated that impaired host autophagy contributes to defective *P. aeruginosa* clearance. Defective cystic fibrosis transmembrane conductance regulator (CFTR) increases reactive oxygen species (ROS) production and tissue transglutaminase 2 activity, leading to Beclin-1 sequestration, p62 accumulation, impaired autophagy, and persistent lung inflammation (Luciani et al., 2010). Consistent with this mechanism, macrophages carrying the F508del-CFTR mutation show impaired uptake and clearance of *P. aeruginosa*, whereas restoration of Beclin-1 and autophagy partially recovers these antibacterial functions (Ferrari et al., 2017).

Biofilm formation represents another bacterial state that may influence autophagy during chronic infection. *P. aeruginosa* biofilms resist antibiotic treatment and immune clearance through extracellular matrix production, metabolic heterogeneity, and altered bacterial physiology. Studies of other bacterial infections have shown that biofilm-associated bacteria can modulate autophagy induction and intracellular trafficking; however, direct evidence demonstrating how *P. aeruginosa* biofilms regulate host autophagic flux remains limited. Current evidence mainly supports an indirect relationship, in which biofilm-associated phenotypes, quorum sensing signals, and chronic adaptation alter bacterial properties sensed by host cells, thereby potentially reshaping autophagic responses.

Collectively, studies involving PAO1, PAK, PA14, PA103, clinical isolates, and chronic infection-adapted strains have progressively expanded our understanding of the interaction between *P. aeruginosa* and host autophagy. Early studies using PAO1 established autophagy as a host defense mechanism, whereas subsequent investigations have shown that diverse clinical strains, bacterial effectors, and toxins may adopt adaptive strategies to redirect or disrupt this response under autophagic pressure.

## 3 Host-protective autophagy

*P. aeruginosa* can invade or establish persistent colonization within host tissues, and different bacterial strains with distinct virulence profiles can induce autophagic responses in various host cell types, including neutrophils, macrophages, epithelial cells, and other cell populations. Host autophagy restricts *P. aeruginosa* infection mainly through two complementary mechanisms. First, antibacterial selective autophagy recognizes and eliminates intracellular bacteria or damaged bacteria-containing compartments, thereby directly reducing bacterial burden. Second, mitophagy removes mitochondria damaged by bacterial virulence factors and infection-associated stress, limiting the accumulation of mitochondrial danger signals and preventing excessive amplification of inflammatory responses. Together, these processes help control bacterial proliferation, reduce infection-associated cellular injury, and maintain tissue homeostasis during *P. aeruginosa* infection. Key host molecules involved in autophagy regulation during *P. aeruginosa* infection are summarized in Table 1.

**Table 1:** Host molecules mediating the interaction between autophagy and *Pseudomonas aeruginosa*.

Molecule	Autophagy function	Function in *P. aeruginosa* infection	Ref.
Initiation			
ULK1–FIP200	Initiation	Supports autophagy and bacterial clearance.	Li et al. (2014)
BECN1–VPS34	Phagophore nucleation	Restoration improves bacterial uptake and clearance in CF macrophages.	Luciani et al. (2010) and Ferrari et al. (2017)
AMPK–mTOR–ULK1	Autophagy initiation	AMPK activation promotes autophagy and bacterial clearance.	Li et al. (2015) and Bone et al. (2021)
Annexin A2 (ANXA2)	Upstream autophagy regulation	Promotes autophagy and bacterial clearance through AKT–mTOR–ULK1/2 signaling.	Li et al. (2015)
Elongation and cargo capture			
ATG5–ATG7	LC3 conjugation	Loss impairs autophagy and bacterial clearance.	Yuan et al. (2012) and Pu et al. (2017)
LC3B	Autophagosome formation	LC3 recruitment to bacteria-containing compartments supports bacterial clearance.	Yuan et al. (2012) and Junkins et al. (2013)
p62/SQSTM1	Selective cargo recognition	Links selective cargo recognition to inflammatory and phagocytic responses.	Pang et al. (2021)
Mitophagy			
PHB2	Mitophagy	Promotes mitophagy and supports antimicrobial defense.	Huang et al. (2020)
PINK1–Parkin	Mitophagy initiation	Promotes mitochondrial quality control during infection.	Lazarou et al. (2015) and Bone et al. (2021)
Terminal degradation and lysosomal function			
TFEB	Lysosome biogenesis	Impaired TFEB activation suppresses autophagy–lysosome function and worsens infection.	Wu et al. (2025)
V-ATPase	Lysosomal degradation	Reduced lysosomal acidification impairs intracellular bacterial processing.	Rodriguez Gonzalez et al. (2024)
Inflammasome			
NLRC4	Inflammasome–autophagy crosstalk	Links bacterial sensing to autophagy-regulated inflammatory responses.	Miao et al. (2008)
NLRP3	Inflammasome–autophagy crosstalk	Autophagy deficiency enhances NLRP3-associated inflammation.	Deng et al. (2016) and Nakahira et al. (2011)

### 3.1 Xenophagy-mediated bacterial clearance

Xenophagy refers to the selective elimination of invading microorganisms or pathogen-damaged compartments by the autophagy machinery. The current concept of xenophagy has been largely established through studies of intracellular pathogens, including *Salmonella enterica*, *Shigella flexneri*, and *Streptococcus pyogenes* (Gutierrez et al., 2004a; Nakagawa et al., 2004). Following bacterial invasion or damage of bacteria-containing membranes, ubiquitin chains can be deposited on bacterial surfaces or surrounding membrane structures, providing recognition signals for selective autophagy receptors.

Several autophagy receptors, including p62/SQSTM1, nuclear dot protein 52 kDa (NDP52), optineurin (OPTN), and Tax1 binding protein 1 (TAX1BP1), contain ubiquitin-binding domains and LC3-interacting regions (LIRs). These receptors simultaneously recognize ubiquitinated microbial structures and recruit LC3/GABARAP proteins to the forming autophagic membrane, thereby linking bacterial cargo with autophagosome formation (Thurston et al., 2009; Wild et al., 2011; Gomes and Dikic, 2014). In addition to ubiquitin-dependent recognition, membrane damage can also initiate xenophagic responses through ubiquitin-independent mechanisms. For example, disruption of pathogen-containing vacuoles exposes luminal glycans to the cytosol, which are detected by galectins. Galectin-8 recognizes damaged vacuolar membranes and recruits NDP52, thereby connecting membrane damage sensing with selective autophagy-mediated bacterial clearance (Thurston et al., 2012).

Although the classical xenophagy pathway has been extensively characterized in several intracellular pathogens, whether *P. aeruginosa* is recognized through an identical ubiquitin-dependent mechanism remains unclear. Current evidence indicates that *P. aeruginosa* infection activates ATG-dependent mechanisms that promote bacterial sequestration and lysosomal delivery. But the precise bacterial signals, ubiquitin ligases, and selective receptors involved in *P. aeruginosa* recognition have not been fully defined, requiring further research.

In *P. aeruginosa* infection, multiple host signaling pathways regulate the recruitment and activation of autophagy machinery. Following uptake by primary alveolar macrophages, *P. aeruginosa* is enclosed within autophagic compartments and induces an increase in LC3-positive puncta, initiating bacterial clearance. Silencing BECN1 or ATG5, both of which are required for autophagosome formation, reduces bactericidal activity, whereas induction of autophagy with rapamycin or interferon-γ enhances bacterial killing (Yuan et al., 2012). *In vivo* studies similarly show that pharmacological induction of autophagy lowers the pulmonary bacterial burden in infected mice, whereas autophagy inhibition increases it (Junkins et al., 2013). *In vitro* and *in vivo* evidence collectively demonstrates that autophagy contributes to intracellular restriction and clearance of *P. aeruginosa*, thereby reducing bacterial burden in the host.

Subsequent studies have further defined the molecular processes by which internalized bacteria are transferred from bacteria-containing compartments to the autophagy–lysosome system. As illustrated in Figure 1, the TLR2–Lyn axis links bacterial uptake and intracellular trafficking with the recruitment of autophagy-related machinery and subsequent delivery to degradative compartments. Following contact with alveolar macrophages, *P. aeruginosa* activates the plasma-membrane receptor TLR2 and the Src-family kinase Lyn, which contributes to bacterial uptake. Lyn regulates phosphoinositide 3-kinase (PI3K)–AKT signaling and cofilin-dependent F-actin remodeling, allowing bacteria-containing phagosomes to move along the cytoskeleton and interact with autophagy-related membrane structures. These events support intracellular transport of ingested bacteria, promote recruitment of ATG12–ATG5 and LC3 to bacteria-containing compartments, and couple this process to Rab5- and Rab7-dependent compartment maturation, enabling bacteria to progressively enter LC3- and LAMP1-positive degradative compartments (Kannan et al., 2006, 2008; Li et al., 2016). Autophagy inhibition or Lyn silencing not only reduces LC3 recruitment around bacteria but also impairs bacterial uptake and subsequent clearance by alveolar macrophages and delays degradation of bacterial proteins within lysosome-related compartments. Consistent with these cellular findings, Lyn-deficient mice show markedly increased susceptibility to pulmonary infection (Li et al., 2016).

**Figure 1:**
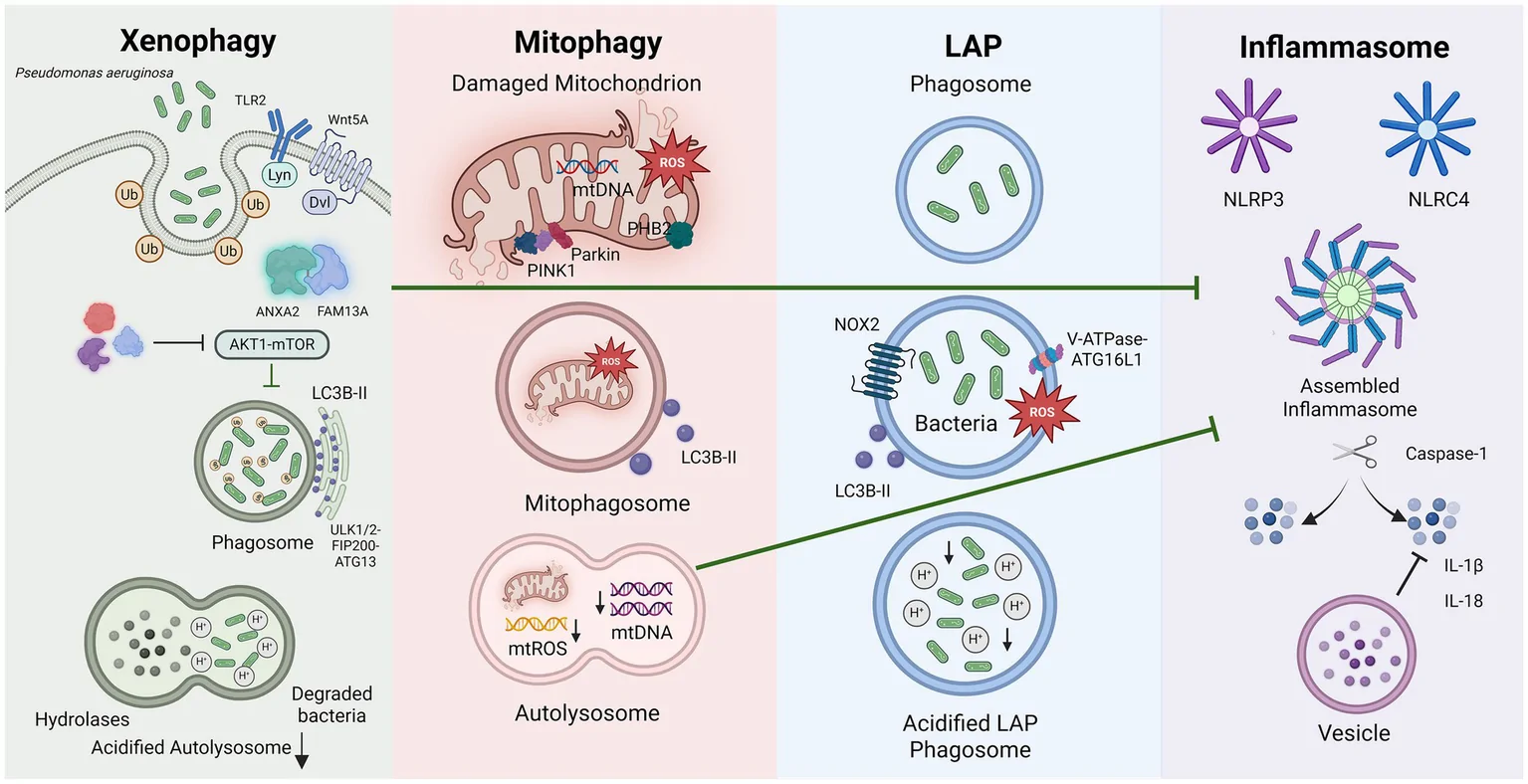
Host-protective autophagy-related responses during *Pseudomonas aeruginosa* infection. The schematic illustrates the major autophagy-related processes involved in host defense against *P. aeruginosa*, including xenophagy, mitophagy, LAP and their interaction with inflammasome signaling. During bacterial uptake, TLR2–Lyn and Wnt5A–Dvl signaling contribute to cytoskeletal remodeling and intracellular trafficking, whereas ANXA2–FAM13A-associated signaling regulates AKT1–mTOR activity and autophagy initiation. Recruitment of LC3 and activation of the ULK1/2–FIP200–ATG13 machinery facilitate the processing of bacteria-containing compartments and their subsequent delivery to acidified degradative compartments. Mitophagy removes infection-damaged mitochondria through PINK1–Parkin-associated and PHB2-dependent pathways, thereby limiting the accumulation of mitochondrial ROS and mtDNA. LAP process involving NOX2-dependent ROS production and V-ATPase–ATG16L1 signaling may promote LC3 lipidation of single-membrane phagosomes and phagosomal acidification, although direct evidence for canonical LAP during *P. aeruginosa* infection remains limited. By reducing intracellular bacterial burden and mitochondrial danger signals, these autophagy-related processes can restrain excessive inflammasome activation and downstream caspase-1-dependent inflammatory responses. ANXA2, annexin A2; Dvl, Disheveled; LAP, LC3-associated phagocytosis; LC3B-II, lipidated microtubule-associated protein 1 light chain 3 beta; mtDNA, mitochondrial DNA; mtROS, mitochondrial reactive oxygen species; NOX2, NADPH oxidase 2; PHB2, prohibitin 2; ROS, reactive oxygen species; TLR2, Toll-like receptor 2; Ub, ubiquitin; V-ATPase, vacuolar H^+^-ATPase; FAM13A, family with sequence similarity 13 member A; FIP200, focal adhesion kinase family interacting protein of 200 kDa; mTOR, mechanistic target of rapamycin; PINK1, PTEN-induced kinase 1; ULK1/2, unc-51-like autophagy-activating kinase 1/2; ATG16L1, autophagy-related 16-like 1. Created by the authors using BioRender.com.

Cytoskeletal remodeling also participates in the interaction between bacteria-containing compartments and the autophagy machinery. Wnt5A signaling activated during *P. aeruginosa* infection promotes Disheveled (Dvl)-dependent actin polymerization through Rac1 activation, while simultaneously enhancing ULK1 activation and LC3 lipidation. This pathway links bacterial internalization-associated cytoskeletal changes with autophagosome formation and contributes to intracellular bacterial restriction (Jati et al., 2018). The ULK1/2–ATG13–FIP200 complex also represents a key regulatory complex involved in infection-induced autophagosome formation. In particular, FIP200 functions as a core scaffold component of the ULK1 initiation complex, and its depletion during *P. aeruginosa* infection reduces autophagic flux and impairs bacterial clearance efficiency (Li et al., 2014). These coordinated cytoskeletal and autophagy-initiation processes are summarized in Figure 1.

Autophagy initiation is further controlled by infection-induced regulation of upstream signaling complexes. Annexin A2 (ANXA2), a membrane-associated protein involved in vesicle trafficking and autophagosome formation, interacts with FAM13A following *P. aeruginosa* infection. The ANXA2–FAM13A axis suppresses AKT1–mTOR signaling, thereby releasing inhibition of the ULK1/2–ATG13–FIP200 complex and promoting autophagosome formation. In Anxa2-deficient mice, impaired macrophage bacterial handling, increased pulmonary bacterial burden, and aggravated inflammatory injury were observed, demonstrating the importance of this pathway in host defense against *P. aeruginosa* (Li et al., 2015).

The importance of this mechanism is further highlighted in cystic fibrosis (CF), where defective autophagy contributes to impaired host defense against chronic *P. aeruginosa* infection. CFTR dysfunction promotes oxidative stress and tissue transglutaminase 2 activation, resulting in Beclin-1 sequestration and impaired autophagy initiation (Luciani et al., 2010). In macrophages carrying the F508del-CFTR mutation, restoration of Beclin-1-dependent autophagy improves bacterial uptake and clearance (Ferrari et al., 2017). CFTR deficiency disrupts the terminal stages of autophagic flux. *P. aeruginosa* challenge impairs autophagosome–lysosome fusion, and elevates lysosomal luminal pH. Consequently, bacteria may become enclosed within phagosomal or autophagy-related vesicles yet remain inadequately degraded because these compartments fail to achieve sufficient acidification (Rodriguez Gonzalez et al., 2024).

Together, these studies indicate that, when *P. aeruginosa* enters host cells, the induced autophagy–lysosome system can enclose bacteria within autophagic membranes, transport bacteria-containing compartments, and deliver them to lysosomes for degradation. Multiple host signaling pathways regulate this clearance process, but an intact and fully functional autophagy–lysosome pathway is required to effectively restrict the intracellular survival of *P. aeruginosa*.

In addition to canonical xenophagy, LC3-associated phagocytosis (LAP) represents another LC3-dependent antimicrobial pathway. Unlike xenophagy, LAP involves direct LC3 lipidation on single-membrane phagosomes rather than formation of a classical double-membrane autophagosome. LAP requires ATG16L1, NADPH oxidase 2 (NOX2), and V-ATPase-dependent phagosomal maturation (Sanjuan et al., 2007; Yang et al., 2012; Martinez et al., 2015). Several observations suggest that LAP-like mechanisms may participate in macrophage responses to *P. aeruginosa*, including LPS-induced TLR signaling, NOX2-dependent oxidative burst, and LC3 recruitment to bacteria-containing phagosomes (Li et al., 2016; Cavinato et al., 2020). However, because LC3 recruitment alone cannot distinguish LAP from other LC3-associated processes, direct evidence demonstrating LAP activation during *P. aeruginosa* infection remains limited. This proposed LAP-like process, including NOX2-dependent ROS production and V-ATPase–ATG16L1-associated phagosomal processing, is illustrated in Figure 1.

### 3.2 Mitophagic surveillance

Unlike xenophagy, which directly targets intracellular pathogens, mitophagy maintains host cellular homeostasis by selectively eliminating damaged mitochondria generated during infection. Under physiological conditions, mitophagy removes dysfunctional mitochondria to maintain mitochondrial quality control. During bacterial infection, however, mitochondrial damage becomes amplified by pathogen-derived toxins, oxidative stress, altered metabolism, and immune activation. Removal of damaged mitochondria prevents excessive accumulation of mitochondrial reactive oxygen species (mtROS), mitochondrial DNA (mtDNA), and other danger-associated molecular patterns, thereby limiting infection-associated cellular injury.

The best-characterized mitophagy pathway is the PINK1–Parkin-dependent mechanism. Loss of mitochondrial membrane potential prevents PINK1 degradation, resulting in its accumulation on the outer mitochondrial membrane and phosphorylation of ubiquitin. Activated Parkin subsequently promotes mitochondrial ubiquitination, allowing autophagy receptors such as OPTN and NDP52 to connect damaged mitochondria with LC3-positive autophagic membranes (Narendra et al., 2008; Koyano et al., 2014; Lazarou et al., 2015). In addition, receptor-mediated pathways involving BNIP3, NIX, FUNDC1, and PHB2 directly interact with LC3 through LC3-interacting regions and mediate mitochondrial clearance independently of Parkin.

Infection with *P. aeruginosa* induces the miR-302/367 cluster, which enhances LC3 processing, reduces mitochondrial ROS accumulation, improves mitochondrial membrane potential, and promotes intracellular bacterial clearance. Mechanistically, prohibitin 2 (PHB2), an inner mitochondrial membrane mitophagy receptor, is required for this response. Following mitochondrial depolarization and outer mitochondrial membrane disruption, PHB2 interacts with LC3 through its LC3-interacting region, facilitating recruitment of damaged mitochondria into autophagic structures. Silencing PHB2 abolishes miR-302/367-mediated activation of mitophagy and bactericidal activity (Huang et al., 2020; Wei et al., 2017). Mitophagy is activated primarily as a response to infection-induced mitochondrial stress. One well-characterized example involves the siderophore pyoverdine. In *Caenorhabditis elegans*, pyoverdine causes intracellular iron depletion, mitochondrial dysfunction, loss of membrane potential, and mitochondrial fragmentation, thereby activating PINK1–Parkin-associated mitophagy. Genetic disruption of autophagy genes (bec-1, lgg-1) or mitophagy-related genes (pink-1, pdr-1) increases susceptibility to pyoverdine toxicity (Kirienko et al., 2013; Kirienko et al., 2015). As summarized in Figure 1, PINK1–Parkin-associated and PHB2-dependent mitophagic pathways contribute to the removal of infection-damaged mitochondria.

Infection-induced mitochondrial damage does not simply accumulate unchecked; rather, it is detected by stress-surveillance programs and subsequently processed and eliminated by the mitophagy machinery. Pyoverdine-induced mitochondrial injury can simultaneously activate stress-responsive transcriptional surveillance and damaged-organelle clearance programs (Kang et al., 2018). Among these, the ethanol and stress response element (ESRE) network mounts a transcriptional response to mitochondrial damage by sensing elevated intracellular reactive oxygen species and redox imbalance, and may be linked to the subsequent initiation and execution of mitophagy. Disruption of the ESRE network markedly reduces host tolerance to pyoverdine toxicity (Tjahjono and Kirienko, 2017; Tjahjono et al., 2020). The mitochondrial unfolded protein response (UPRmt), in turn, uses ATFS-1 to sense impaired mitochondrial protein import and disrupted proteostasis, thereby inducing genes involved in molecular chaperoning, proteolysis, detoxification, and metabolic remodeling (Deng et al., 2019). ATFS-1-mediated UPRmt can functionally coordinate with FNDC-1-mediated mitophagy, integrating mitochondrial adaptation with selective organelle clearance to shape the cellular response to infection-associated stress (Lim et al., 2021). *P. aeruginosa* infection can specifically induce expression of the basic leucine zipper (bZIP) protein ZIP-3, which suppresses UPRmt activation and thereby weakens the host defensive adaptation that would otherwise be reinforced by mitochondrial stress signaling (Deng et al., 2019).

Thus, mitophagy-mediated removal of damaged mitochondria during *Pseudomonas aeruginosa* infection represents an innate defense mechanism that not only helps eliminate bacteria but also enhances tolerance to damage rather than serving only basal organelle homeostasis. Notably, if terminal autophagic flux is impaired, dysfunctional mitochondria persist and continue to release mitochondrial DNA and mtROS, which may in turn activate the inflammasome pathways discussed below.

### 3.3 Inflammasome control

Autophagy during *P. aeruginosa* infection not only contributes to the clearance of intracellular bacteria and damaged mitochondria but also regulates inflammasome activation, thereby promoting pathogen control while limiting inflammatory injury (Deretic and Levine, 2018).

Inflammasome activation and associated tissue injury are well documented during *P. aeruginosa* infection. Cytosolic flagellin and the structural components of the type III secretion system (T3SS) can be recognized by the NAIP–NLRC4 inflammasome, leading to caspase-1 activation, interleukin-1β (IL-1β) maturation, and pyroptosis (Miao et al., 2008). Infection-associated mitochondrial injury may further enhance NLRC4-related inflammatory responses (Jabir et al., 2015). In CF airway epithelial cells, *P. aeruginosa* and its flagellin increase mitochondrial Ca^2+^ loading and mitochondrial dysfunction and enhance NLRP3 inflammasome activation and maturation of IL-1β and interleukin-18 (IL-18) through mitochondrial calcium uniporter-dependent signaling (Rimessi et al., 2015).

Autophagy can suppress the activation of inflammasomes and their damaging effects following infection. First, xenophagy reduces the intracellular bacterial burden and limits persistent cytosolic exposure to lipopolysaccharide, flagellin, secretion-system components, and other pathogen-associated molecules. For bacteria capable of remaining within host cells, successful completion of cargo recognition, autophagic sequestration, and lysosomal degradation progressively reduces pathogen-derived inflammatory stimulation. Second, autophagy and mitophagy remove mitochondria with reduced membrane potential and impaired electron transport, thereby limiting cytosolic accumulation of mtROS, oxidized mitochondrial DNA, and other mitochondrial danger signals. Because these signals can promote NLRP3 inflammasome assembly and activation, timely removal of damaged mitochondria reduces the intensity and duration of NLRP3 signaling (Nakahira et al., 2011; Jabir et al., 2015).

Autophagy can also act directly on inflammasome complexes. Activated and ubiquitinated NLRP3 can be recognized by the selective autophagy receptor p62, delivered to autophagosomes, and degraded in lysosomes. This process reduces the abundance of active NLRP3 and promotes termination of inflammatory signaling rather than merely preventing the formation of new inflammasomes (Biasizzo and Kopitar-Jerala, 2020). Autophagy can therefore restrain inflammasome activation both by removing upstream sources of danger signals and by degrading signaling complexes that have already formed. These complementary mechanisms by which autophagic bacterial clearance and mitochondrial quality control restrain inflammasome signaling are summarized in Figure 1.

Loss of autophagy proteins can markedly amplify inflammasome responses. ATG16L1 deficiency enhances caspase-1 activation and LPS-induced maturation and release of IL-1β through a TIR-domain-containing adapter-inducing interferon-β (TRIF)-dependent mechanism, indicating that an intact autophagy apparatus exerts negative control over inflammasome signaling (Saitoh et al., 2008). During *P. aeruginosa* infection, myeloid Atg7 deficiency increases IL-1β production and macrophage pyroptosis and is accompanied by greater bacterial dissemination and increased mortality in mice (Pu et al., 2017). Loss of epithelial Atg5 can also enhance noncanonical caspase-11 activation and mitochondrial DNA release, thereby amplifying inflammation in neighboring macrophages through cGAS–STING–NLRP3 signaling. Autophagy failure in one cellular compartment may therefore propagate inflammatory injury to another (Wang et al., 2025).

It should be noted that the roles of inflammasomes and pyroptosis during *Pseudomonas aeruginosa* infection remain controversial. For example, NAIP–NLRC4-dependent macrophage pyroptosis has been reported to trigger pore-induced intracellular traps (PITs), which subsequently enable neighboring neutrophils to eliminate bacteria captured within these traps (Jorgensen et al., 2016; Santoni et al., 2022). In contrast, flagellin-driven NLRC4/TLR5–caspase-1-dependent pyroptosis may impair bacterial clearance and exacerbate acute lung injury (Ryu et al., 2017). Furthermore, the interaction between autophagy and pyroptosis is not always inhibitory. Outer membrane vesicles derived from *P. aeruginosa* can simultaneously activate autophagy and inflammasome-related signaling pathways. In monocytes, autophagy may promote pyroptosis-associated inflammatory responses (Ge et al., 2025b), whereas NLRP3 activation in airway epithelial cells may exert feedback inhibition on autophagy (Ge et al., 2025a).

## 4 Bacterial countermeasures against host autophagy

Under the selective pressure imposed by host autophagy-mediated defense, *P. aeruginosa* has evolved strategies to counteract this response. Through secreted effector proteins, quorum-sensing molecules, and modulation of infection-induced host signaling pathways, *P. aeruginosa* can interfere with multiple stages of the autophagy–lysosome pathway, thereby suppressing or redirecting autophagic processes to promote its own survival.

### 4.1 Autophagy suppression

#### 4.1.1 Inhibition of autophagosome formation

ExoS is a bifunctional effector delivered by the *P. aeruginosa* T3SS and possesses both GTPase-activating protein and ADP-ribosyltransferase activities. Its ADP-ribosyltransferase domain inhibits the lipid-kinase activity of the ATG14L-associated VPS34 complex, reduces phosphatidylinositol 3-phosphate production, and thereby impairs isolation-membrane nucleation and early autophagosome formation. Because PI3P is a key lipid signal for recruiting WIPI proteins, positioning ATG complexes, and promoting isolation-membrane elongation, inhibition of VPS34 by ExoS acts upstream of autophagosome biogenesis and may allow internalized bacteria to escape before entering a complete degradative pathway (Rao et al., 2021). In ATG7-deficient airway epithelial cells, intracellular survival of an ExoS-deficient strain increased markedly, whereas the effect on a strain expressing functional ExoS was relatively limited. This indicates that ExoS enables bacteria to evade an otherwise antibacterial ATG7-dependent autophagic defense (Rao et al., 2021) by targeting the autophagic membrane-nucleation machinery and reducing host clearance of *P. aeruginosa*.

A recent study identified another *Pseudomonas aeruginosa* factor, Pec1, which is related to autophagy inhibition. In alveolar macrophages, Pec1 reduces integrin β3 expression, lowers miR-155 levels, and enhances PI3K–AKT–mTOR signaling. These changes are accompanied by reduced formation of autophagy-related structures and impaired intracellular bacterial clearance. In animal experiments, Pec1 expression was associated with increased pulmonary bacterial burden and tissue injury. Its abundance in clinical sputum samples also correlated with *P. aeruginosa* burden (Jin et al., 2024). Unlike ExoS, which directly inhibits VPS34 lipid-kinase activity, Pec1 mainly restricts autophagy initiation indirectly through the host integrin β3–miR-155–PI3K–AKT–mTOR pathway. These findings represent two different ways in which bacteria interfere with formation of autophagosomes: one involves an effector acting directly on the core autophagy machinery to produce rapid and relatively well-defined enzymatic inhibition, whereas the other alters host-receptor and noncoding-RNA signaling to reset the threshold for autophagy initiation.

#### 4.1.2 Lysosomal dysfunction

The effects of ExoS on host degradative pathways extend beyond autophagosome formation. In airway epithelial cells, bacteria lacking ExoS or its ADP-ribosyltransferase activity enter LAMP-associated, LysoTracker-positive acidic compartments more readily and show reduced replication within these compartments. Pharmacological inhibition of V-ATPase-dependent acidification partially restores intracellular replication of the mutant strains. These results indicate that ExoS reduces bacterial entry into, or retention within, acidic bactericidal compartments and promotes intracellular replication (Heimer et al., 2013). Subsequent work further showed that ExoS can promote escape from bacteria-containing vacuoles and generate heterogeneous intracellular bacterial fates, with some bacteria entering the cytosol and undergoing replication, whereas others remaining within membrane-bound compartments. ExoS may therefore remodel the intracellular niche not only by limiting autophagic membrane formation but also by altering vacuolar acidification, membrane integrity, and compartmental transitions (Kroken et al., 2022).

In addition to the direct alteration of autophagic lysosomal function by bacterial effector proteins, infection can also inhibit lysosomal biogenesis through host calpain-1 (CAPN1). Following *P. aeruginosa* infection, CAPN1 expression increases, and CAPN1 interacts with TFEB, a transcription factor involved in lysosomal biogenesis. This interaction restricts TFEB nuclear translocation and reduces the expression of lysosome- and autolysosome-related genes. These changes are accompanied by reduced lysosomal abundance and impaired formation of autophagic compartments. Genetic loss of CAPN1 restores TFEB nuclear localization, rescues autophagic flux and lysosomal function, and attenuates infection-associated lung injury (Wu et al., 2025).

#### 4.1.3 Epigenetic and metabolic suppression of autophagy

Compared with the direct enzymatic activity of T3SS effectors, the quorum-sensing-associated metabolite 2-aminoacetophenone (2-AA) modulates autophagy through epigenetic and metabolic reprogramming, establishing a more prolonged tolerant state in macrophages. Treatment with 2-AA, or infection with *P. aeruginosa* retaining normal 2-AA production, reduces ULK1 and BECN1 expression, decreases LC3-associated structures, and impairs macrophage clearance of intracellular bacteria (Chakraborty et al., 2023). Mechanistically, 2-AA enhances histone deacetylase 1 (HDAC1)-dependent deacetylation and lowers H3K18 acetylation at the BECN1 and SCD1 promoters, shifting the corresponding genes from a transcriptionally active to a repressed state. Reduced BECN1 expression limits VPS34-dependent autophagic membrane nucleation, whereas reduced stearoyl-CoA desaturase 1 (SCD1) expression affects the production of monounsaturated fatty acids and membrane lipids. Accordingly, repression of BECN1 and SCD1 is associated with reduced autophagosome formation. HDAC1 knockdown alleviates these changes, while supplementation with palmitoyl-CoA and stearoyl-CoA partially restores macrophage bacterial clearance (Chakraborty et al., 2023).

The effects of 2-AA also involve inflammatory transcription. Related studies show that, through HDAC1-mediated epigenetic reprogramming, 2-AA reduces H3K18 acetylation at pro-inflammatory gene loci, increases the repressive nuclear factor-κB (NF-κB) p50–HDAC1 complex, and weakens both the interaction between p65 and CBP/p300 and p65 acetylation, causing macrophages to display a weaker inflammatory response to subsequent infectious stimulation (Bandyopadhaya et al., 2016, 2017). Although this hyporesponsive state may limit inflammatory injury to some extent, it may also reduce the host’s ability to recognize and clear bacteria promptly, thereby creating an immune-tolerant environment favorable to chronic persistent infection.

In related studies, 2-AA alters mitochondrial bioenergetics and pyruvate metabolism through PPARGC1A/ESRRA-related regulation, reducing oxidative metabolic capacity and the energy available to host cells for antimicrobial responses (Chakraborty et al., 2024). Autophagy is highly dependent on energy, membrane lipids, and organelle trafficking. Reduced mitochondrial bioenergetics may therefore limit autophagosome formation and also weaken other antimicrobial functions, including phagocytosis, the oxidative burst, and inflammatory-cytokine synthesis.

Taken together, the suppression of host autophagy by *P. aeruginosa* is not confined to a single virulence factor or a fixed molecular target as summarized in Figure 2. Instead, it affects multiple stages, ranging from transcriptional regulation, autophagy initiation, and membrane-lipid and energy supply to the maturation of bacteria-containing compartments and lysosomal degradation. Consequently, internalized bacteria are less likely to enter autophagy-related pathways and, once recruited, may also be less efficiently eliminated.

**Figure 2:**
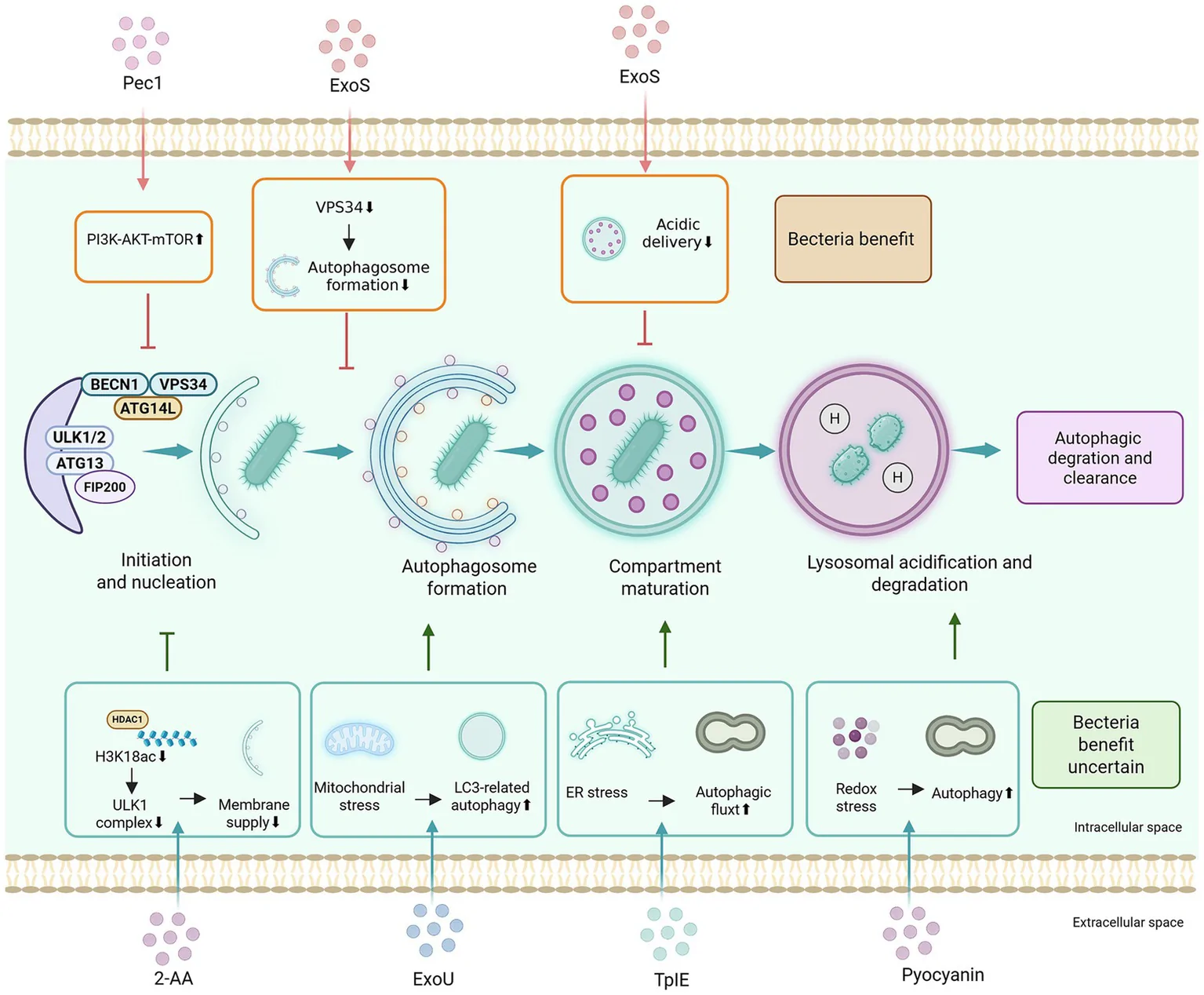
*Pseudomonas aeruginosa* interference with host autophagy at multiple stages of the autophagy–lysosome pathway. The schematic summarizes how *P. aeruginosa* virulence-associated factors suppress, induce, or redirect host autophagy from initiation and autophagosome formation to compartment maturation and lysosomal degradation. Pec1 activates PI3K–AKT–mTOR signaling and suppresses autophagy initiation, whereas the T3SS effector ExoS inhibits VPS34 activity and autophagosome formation. ExoS also reduces the entry or retention of intracellular bacteria within acidic degradative compartments, thereby favoring bacterial persistence. The quorum-sensing metabolite 2-aminoacetophenone (2-AA) induces HDAC1-dependent epigenetic reprogramming, reduces H3K18 acetylation, and suppresses autophagy-related signaling and membrane supply. In contrast, several bacterial factors induce stress-associated autophagic responses. ExoU induces mitochondrial stress and LC3-associated autophagic responses, TplE activates ER stress and increases autophagic flux, and pyocyanin induces redox stress and autophagy. However, these responses do not uniformly favor bacterial persistence. 2-AA, 2-aminoacetophenone; AKT, protein kinase B; BECN1, Beclin 1; ER, endoplasmic reticulum; HDAC1, histone deacetylase 1; H3K18ac, histone H3 lysine 18 acetylation; LC3, microtubule-associated protein 1 light chain 3; mTOR, mechanistic target of rapamycin; PI3K, phosphoinositide 3-kinase; T3SS, type III secretion system; ULK1/2, unc-51-like autophagy-activating kinase 1/2; VPS34, class III phosphatidylinositol 3-kinase; ATG14L, autophagy-related 14-like; ATG13, autophagy-related 13; FIP200, focal adhesion kinase family interacting protein of 200 kDa. Created by the authors using BioRender.com.

### 4.2 Autophagy induction and redirection

Inhibiting autophagy is not the only strategy employed by *P. aeruginosa* to initiate and sustain infection by evading host cell clearance. Certain virulence factors of *P. aeruginosa* and infection-related stressors can significantly induce autophagy-related responses; however, this enhanced autophagy does not necessarily lead to effective pathogen degradation. Instead, it may be accompanied by alterations in phagocytic capacity, oxidative killing, inflammatory signaling, or host cell homeostasis.

Direct evidence indicates that *Pseudomonas aeruginosa* infection induces autophagy in both murine and human macrophages, thereby enhancing macrophage bactericidal activity. However, further stimulation of autophagy by rapamycin treatment or nutrient starvation reduces the expression of certain phagocytic receptors, decreases bacterial uptake, and is accompanied by diminished production of reactive oxygen and nitrogen intermediates, ultimately impairing intracellular bacterial killing (Wu et al., 2016). Early growth response 1 (EGR1) may serve as a critical regulator underlying the dissociation between autophagy activation and macrophage phagocytic function. Following acute pulmonary infection with *P. aeruginosa*, EGR1 expression is induced and promotes the transcription of autophagy-related genes, including LC3A, LC3B, and ATG5, leading to enhanced degradation of p62. Beyond its role as an autophagic substrate and selective autophagy receptor, p62 also contributes to the stabilization and activation of NRF2. Therefore, excessive reduction of p62 levels weakens NRF2 signaling and decreases the expression of scavenger receptors such as MARCO and MSR1, thereby limiting macrophage uptake of *P. aeruginosa*. Pharmacological activation of autophagy with rapamycin attenuates the enhanced phagocytic activity caused by EGR1 silencing, whereas chloroquine partially restores the phagocytic defects induced by EGR1 overexpression (Pang et al., 2021).

Indirect evidence that autophagy activation may impair bacterial clearance is also provided by host β-defensin 2 and β-defensin 3. Following *P. aeruginosa* infection, the expression of these antimicrobial peptides is increased. They suppress macrophage autophagy by downregulating EGR1 and c-FOS, while simultaneously enhancing bacterial phagocytosis and phagolysosome formation, thereby promoting *P. aeruginosa* clearance (Wu et al., 2018).

In addition, the quorum-sensing molecule N-(3-oxo-dodecanoyl)-L-homoserine lactone (3-oxo-C12-HSL) produced by *P. aeruginosa* increases cytosolic Ca^2+^ levels in macrophages, resulting in mitochondrial dysfunction, upregulation of key autophagy-related molecules, and enhanced formation of autophagic vacuoles. However, the resulting mitochondrial damage and autophagic activation further promote macrophage apoptosis, thereby compromising sustained macrophage function (Kushwaha et al., 2022).

Therefore, during *P. aeruginosa* infection, unlike some other bacterial infections in which autophagosome formation and delivery to degradative compartments effectively contribute to bacterial elimination (Nakagawa et al., 2004; Ligeon et al., 2014; Pavlik et al., 2024), autophagy activation and phagocytic clearance do not always occur in parallel. Excessive or dysregulated autophagy may instead impair macrophage antimicrobial capacity by reducing bacterial uptake, disrupting cellular metabolism, or promoting apoptosis.

Inflammasome activation and assembly may also contribute to the modulation of autophagy by *P. aeruginosa*, thereby influencing bacterial persistence during infection. In human macrophages, *P. aeruginosa* infection induces NLRP3 and ASC expression together with a caspase-1-dependent autophagy-associated response. Silencing inflammasome components decreases LC3-II accumulation and enhances intracellular bacterial clearance, whereas depletion of ATG7 or BECN1 produces similar effects (Deng et al., 2016). In addition, the T3SS effector ExoU localizes to mitochondria or mitochondria-associated membranes, where it disrupts membrane lipids and induces oxidative stress and mitochondrial damage, while simultaneously triggering stress-associated autophagy. Rather than promoting host defense, this autophagic response suppresses NLRC4-dependent caspase-1 activation and IL-1β release, thereby limiting potentially protective inflammatory danger signals and facilitating immune evasion (Hardy et al., 2022).

Notably, pyocyanin, a virulence factor secreted by *P. aeruginosa*, elicits autophagic responses with divergent functional outcomes. In airway epithelial cells, pyocyanin induces autophagy through activation of the GCN2–eIF2α–ATF4 axis of the integrated stress response. In macrophages, it modulates ULK1 transcription and autophagic activity by altering the acetylation status of HMGN2 and histone H3 lysine 27 (H3K27). Strains deficient in the pyocyanin biosynthetic gene *phzM* exhibit attenuated autophagy induction yet display increased bacterial burdens and elevated host mortality (Yang et al., 2016; Du et al., 2021). These observations challenge the oversimplified notion that virulence factor-induced autophagy usually benefits bacterial survival, and instead support the view that pyocyanin-triggered autophagy contributes to host defense or tissue tolerance, despite being a classical virulence-associated molecule. In human corneal epithelial cells, T3SS toxins can also induce BECN1 and autophagy, and reduce intracellular bacterial loads of certain clinical strains. In contrast, T3SS-deficient strains exhibit weaker induction of autophagy but show stronger intracellular replication capacity (Mohankumar et al., 2018). These intriguing observations suggest that while some virulence factors cause cellular damage and trigger autophagy, the resulting host response may still restrict bacterial growth. *P. aeruginosa* can also trigger stress-associated autophagy via endoplasmic reticulum (ER) stress. Specifically, the H2-T6SS effector TplE is translocated into host cells, targets the ER membrane, disrupts its structural integrity, activates the IRE1α–XBP1 branch of the unfolded protein response (UPR), and consequently enhances LC3 lipidation and autophagic flux (Jiang et al., 2016). However, genetic or pharmacological inhibition of TplE-induced autophagy has not been demonstrated to reduce bacterial burden or mitigate T6SS-dependent virulence *in vivo* or in relevant infection models. As illustrated in Figure 2, ExoU, TplE, and pyocyanin induce stress-associated autophagic responses, although their consequences for bacterial persistence remain uncertain.

Overall, in addition to suppressing autophagy to escape elimination, *P. aeruginosa* may also induce and redirect autophagy, transforming this pathway from a host defense mechanism into a cellular response that favors bacterial survival, persistent infection, and tissue damage.

### 4.3 Factors influencing the direction of autophagy regulation

Despite extensive research, a unified explanation applicable across all models remains lacking regarding whether autophagy acts as a host-protective mechanism or indirectly favors bacterial persistence and injury following *P. aeruginosa* infection. Moreover, the factors influencing the direction of this effect are complex and difficult to resolve. First, it is crucial to distinguish between whether autophagy is induced and whether autophagy benefits the host, as *P. aeruginosa* and its virulence factors can both suppress and induce autophagy, and the occurrence of autophagy and accumulation of related structures do not necessarily translate into bacterial killing. Second, increased formation of autophagosomes does not guarantee enhanced autophagic flux; whether bacteria are selectively captured and delivered to acidified lysosomes, and whether heightened autophagy comes at the expense of other immune functions such as phagocytosis and oxidative killing may be critical in determining the net effect of autophagy during *P. aeruginosa* infection. Furthermore, the strain composition and virulence factor profile of *P. aeruginosa* may alter the autophagy signals received by host cells. For example, virulence effector proteins ExoS and metabolite 2-AA evade and inhibit cellular autophagy, whereas ExoU, TplE, and pyocyanin primarily induce stress-associated autophagy through mitochondrial damage, endoplasmic reticulum stress, or redox imbalance, resulting in distinct biological consequences. Finally, the direction of autophagy cannot be assessed independently from inflammatory networks and cell fate. Xenophagy and mitophagy may limit excessive inflammasome activation by reducing intracellular bacterial and mitochondrial danger signals, but suppression of inflammation does not necessarily equate to host benefit. Whether autophagy-mediated clearance of bacteria and damaged organelles outweighs the costs associated with pyroptosis, inflammatory spread, and impaired phagocytic function ultimately determines whether there is a net beneficial effect.

## 5 Therapeutic targeting of autophagy

Unlike conventional antimicrobial strategies that directly target bacterial viability, host-directed therapy aims to enhance endogenous defense mechanisms and reduce infection-associated tissue injury. The autophagy–lysosome pathway represents an attractive therapeutic target during *P. aeruginosa* infection because it participates in intracellular bacterial control, mitochondrial quality maintenance, and regulation of excessive inflammatory responses. However, increasing evidence indicates that autophagy is not intrinsically protective in all infection settings. Furthermore, robust preclinical or clinical evidence supporting the therapeutic efficacy of autophagy modulation specifically against *P. aeruginosa* infection remains scarce.

### 5.1 Pharmacological modulation

Pharmacological manipulation of autophagy has primarily focused on promoting autophagy initiation, particularly through regulation of the mTOR and AMP-activated protein kinase (AMPK) signaling pathways. Rapamycin, an inhibitor of mTORC1, is one of the most extensively investigated autophagy-inducing compounds in *P. aeruginosa* infection models. By releasing mTORC1-mediated inhibition of the ULK1 initiation complex, rapamycin enhances autophagosome formation. In alveolar macrophages, rapamycin treatment increases autophagy-associated bacterial clearance following *P. aeruginosa* infection, and pharmacological activation of autophagy reduces bacterial burden in murine models of acute pulmonary infection (Yuan et al., 2012; Junkins et al., 2013). Similar protective effects have also been observed in diabetic wound infection models, where rapamycin improves autophagy-associated antibacterial responses and accelerates bacterial elimination (Xu et al., 2020).

Nevertheless, the therapeutic potential of rapamycin requires careful interpretation because autophagy activation does not always translate into improved antimicrobial immunity. Sustained activation of autophagy or nutrient deprivation-induced autophagy may alter macrophage functional states by reducing phagocytic receptor expression and impairing reactive oxygen and nitrogen species production, thereby weakening bacterial killing capacity (Wu et al., 2016). Moreover, excessive activation of bulk autophagy may not selectively enhance xenophagic degradation of bacteria and could potentially interfere with other immune processes.

AMPK activation represents another approach to regulate autophagy-associated host defense. In a macrophage model of *P. aeruginosa* PAK infection, pharmacological activation of AMPK with 5-aminoimidazole-4-carboxamide ribonucleoside (AICAR) or Metformin enhanced LC3-associated autophagic responses and promoted intracellular bacterial killing. Importantly, direct AMPK agonists, including A-769662 and MK-8722, similarly enhanced bacterial clearance in macrophage models, suggesting that AMPK-dependent regulation of autophagy may represent an alternative strategy to improve antimicrobial function (Bone et al., 2021). Interferon-γ also enhances macrophage activation and promotes autophagy-associated bacterial clearance, further supporting the potential of autophagy to restore antimicrobial capacity (Yuan et al., 2012).

Beyond enhancing autophagy initiation, restoring defects in the autophagy–lysosome pathway represents a potential therapeutic strategy. TFEB, a master regulator of lysosomal biogenesis, has emerged as a candidate target because it coordinates lysosomal formation and autophagic degradation. Recent studies demonstrated that *P. aeruginosa* PAK infection suppresses TFEB activity through CAPN1-dependent mechanisms, resulting in reduced autophagy-associated markers and impaired lysosomal function, whereas TFEB overexpression restores autophagic activity and attenuates inflammatory responses (Wu et al., 2025). In addition, impairment of TFEB-mediated autophagy contributes to defective clearance of PAO1 in macrophages under cigarette smoke exposure conditions, suggesting that maintaining TFEB activity may support host antibacterial defense (Bodas et al., 2019). Pharmacological strategies targeting lysosomal function are also of interest. Because V-ATPase-dependent acidification is essential for lysosomal activity, restoration of lysosomal proton transport may theoretically improve the degradation capacity of autophagic compartments. However, direct evidence demonstrating that V-ATPase-targeting approaches improve *P. aeruginosa* infection outcomes is currently lacking.

Restoration of autophagy and lysosome is particularly relevant to CF, where CFTR deficiency causes autophagic blockade and inadequate terminal lysosomal function, leaving pathogens captured but insufficiently degraded and thereby favoring chronic colonization. Cysteamine restores BECN1 availability and autophagic flux in F508del-CFTR macrophages and improves *P. aeruginosa* clearance (Ferrari et al., 2017). Combined treatment with amiodarone and cysteamine synergistically induces autophagy in F508del-CFTR epithelial cells (Zhang et al., 2018), and these agents have been considered potential candidates for autophagy-directed therapy in CF lung disease (Junkins et al., 2014). The approved CFTR modulators tezacaftor/ivacaftor can also restore autolysosomal acidification and improve bacterial clearance in CF macrophages. However, the relevant infection experiments were performed with *Burkholderia* species, and applicability to *P. aeruginosa* remains to be established (Badr et al., 2022).

Another emerging strategy is to interfere with bacterial mechanisms that disrupt host autophagy. Rather than directly activating host pathways, targeting *P. aeruginosa* virulence factors may preserve endogenous autophagic defense. For example, T3SS effectors such as ExoS and ExoU interfere with host cellular processes, including vesicular trafficking, mitochondrial integrity, and inflammatory signaling. Inhibiting T3SS activity or neutralizing specific effectors may therefore indirectly maintain autophagy-related antimicrobial functions. Although direct evidence connecting T3SS inhibition with restoration of autophagy during *P. aeruginosa* infection remains limited, targeting bacterial factors that manipulate host pathways represents an attractive complementary approach.

Collectively, current evidence suggests that autophagy-targeted therapy should not be restricted to nonspecific induction of autophagy. More precise strategies that restore defective autophagy–lysosome processing, enhance lysosomal function, or prevent bacterial interference with host pathways may provide greater therapeutic potential.

### 5.2 Natural products and bioactive compounds

Natural products have attracted attention as potential host-directed therapeutics because of their broad biological activities, including regulation of inflammation, oxidative stress, bacterial virulence, and autophagy-related pathways. However, direct evidence demonstrating that natural compounds protect against *P. aeruginosa* infection through autophagy modulation remains limited. Among currently available studies, CoB1, Iraqi propolis, and selenomethionine (Se-Met) represent compounds with relatively direct evidence linking their effects to autophagy-associated responses.

CoB1, a cochlioquinone B derivative isolated from the endophytic fungus *Bipolaris sorokiniana* associated with *Salvia miltiorrhiza*, is one of the few natural compounds directly investigated in *P. aeruginosa* infection models. In alveolar macrophages and mouse pulmonary infection models, CoB1 enhances protective autophagy, promotes bacterial clearance, reduces inflammatory injury, and improves survival. Mechanistically, CoB1 regulates the PAK1–AKT–mTOR pathway, thereby relieving mTOR-mediated inhibition of autophagy initiation (Zhu et al., 2020).

Iraqi propolis has also been investigated in *P. aeruginosa* infection models. Unlike compounds that primarily enhance bacterial clearance, propolis appears to exert protective effects mainly through modulation of excessive inflammatory responses. In bone marrow-derived macrophages and mouse models, propolis reduces NLRC4 inflammasome activation, caspase-1 activity, and IL-1β production following *P. aeruginosa* infection, thereby limiting tissue injury (Jabir et al., 2018). However, whether these effects are directly mediated through autophagy modulation remains to be fully established.

Selenomethionine (Se-Met) represents another compound linking autophagy regulation with protection against *P. aeruginosa* infection. Se-Met treatment inhibits PAK1 expression, suppresses AKT–mTOR signaling, and promotes autophagosome formation in alveolar macrophages. In mouse infection models, Se-Met reduces lung injury, bacterial dissemination, and inflammatory responses while improving survival (Nie et al., 2025).

Other natural compounds, including baicalin, curcumin, and quercetin, have demonstrated anti-biofilm, antivirulence, or anti-inflammatory effects against *P. aeruginosa*, and some have been shown to regulate autophagy in other infection models. Representative interventions targeting autophagy during *P. aeruginosa* infection are summarized in Table 2.

**Table 2:** Interventions modulating autophagy in *Pseudomonas aeruginosa* infection.

Agent	Autophagy target	Model	Main finding	Reference
Autophagy induction				
Rapamycin	mTORC1–ULK1	Macrophages; mouse lung infection	Enhances bacterial clearance, although sustained induction may impair macrophage uptake.	Yuan et al. (2012), Junkins et al. (2013), and Wu et al. (2016)
Metformin	AMPK–ULK1/BECN1	Macrophages; post-sepsis mouse model	Enhances autophagy and bacterial clearance.	Bone et al. (2021)
AICAR	AMPK	Macrophages	Enhances Parkin-independent bacterial killing.	Bone et al. (2021)
IFN-γ	Autophagy activation	Alveolar macrophages	Enhances bacterial killing.	Yuan et al. (2012)
CoB1	PAK1–AKT–mTOR	Macrophages; mouse lung infection	Promotes autophagy and bacterial clearance.	Zhu et al. (2020)
Se-Met	PAK1–AKT–mTOR	Macrophages; mouse lung infection	Promotes autophagy and improves infection outcome.	Nie et al. (2025)
Iraqi propolis	IL-1β/NLRC4 degradation	BMDMs; mouse infection	Promotes autophagic inflammasome clearance.	Jabir et al. (2018)
Autophagy restoration				
Cysteamine	TG2–BECN1	CF macrophages	Restores autophagy and bacterial clearance.	Ferrari et al. (2017)
Amiodarone + cysteamine	CFTR proteostasis	CF epithelial cells	Restores autophagy-related proteostasis^†^	Zhang et al. (2018)
Tezacaftor + ivacaftor	Autolysosomal acidification	CF macrophages	Restores lysosomal acidification and bacterial clearance^†^	Badr et al. (2022)

Induction denotes enhancement of autophagy signaling, whereas restoration refers to correction of an impaired autophagy–lysosome pathway.

^†^ Direct *P. aeruginosa*-specific evidence linking the intervention to autophagy-mediated bacterial clearance is limited.

## 6 Conclusions and future perspectives

During *P. aeruginosa* infection, autophagy appears to have a complex, double-edged role that may be related to the bacterial strain, virulence factors, stage of infection, and host condition. More importantly, autophagy is a dynamic defense system comprising consecutive stages of cargo recognition, membrane formation, compartment maturation, lysosomal degradation, and immune functional output. Its functional direction is therefore also constrained by whether these stages are successfully connected.

The preceding sections have reviewed the available studies on *P. aeruginosa* and autophagy and examined the principles and mechanisms underlying host-protective autophagy and bacterial counteraction of autophagy. Host cells can restrict intracellular bacteria by increasing the efficiency of cargo recognition, membrane sequestration, and lysosomal degradation. Conversely, *P. aeruginosa* and its virulence factors can act on the ULK complex, the BECN1–VPS34 nucleation machinery, LC3 lipidation, the maturation of bacteria-containing compartments, or lysosomal acidification, thereby suppressing, redirecting, or otherwise altering autophagic flux at different stages.

Given the limitations of the current evidence, any attempt to therapeutically target autophagy during *P. aeruginosa* infection must account for the complexity of the pathway and the multiple steps required to maintain intact autophagic flux. From a translational perspective, autophagy modulation is more appropriately considered an adjunct to antibiotics than a replacement for antimicrobial treatment. Restoring the clearance capacity of host cells may reduce bacterial persistence and dissemination and improve antibiotic activity against intracellular bacteria or biofilm-associated infections.

Overall, the relationship between *P. aeruginosa* and autophagy should not be reduced to a struggle over a single molecular switch. Rather, it represents a dynamic competition between the host and the pathogen across multiple checkpoints of the autophagy–lysosome pathway.

## References

[ref1] Antimicrobial Resistance Collaborators (2022). Global burden of bacterial antimicrobial resistance in 2019: a systematic analysis. Lancet 399, 629–655. doi: 10.1016/S0140-6736(21)02724-0, 37500506

[ref2] Badr A. Eltobgy M. Krause K. Hamilton K. Estfanous S. Daily K. P. . (2022). CFTR modulators restore acidification of autophago-lysosomes and bacterial clearance in cystic fibrosis macrophages. Front. Cell. Infect. Microbiol. 12:819554. doi: 10.3389/fcimb.2022.819554, 35252032 PMC8890004

[ref3] Bandyopadhaya A. Tsurumi A. Maura D. Jeffrey K. L. Rahme L. G. (2016). A quorum-sensing signal promotes host tolerance training through HDAC1-mediated epigenetic reprogramming. Nat. Microbiol. 1:16174. doi: 10.1038/nmicrobiol.2016.174, 27694949 PMC5066596

[ref4] Bandyopadhaya A. Tsurumi A. Rahme L. G. (2017). NF-κBp50 and HDAC1 interaction is implicated in the host tolerance to infection mediated by the bacterial quorum sensing signal 2-aminoacetophenone. Front. Microbiol. 8:1211. doi: 10.3389/fmicb.2017.01211, 28713342 PMC5492500

[ref5] Barbieri J. T. Sun J. (2004). *Pseudomonas aeruginosa* ExoS and ExoT. Rev. Physiol. Biochem. Pharmacol. 152, 79–92. doi: 10.1007/s10254-004-0031-7, 15375697

[ref6] Biasizzo M. Kopitar-Jerala N. (2020). Interplay between NLRP3 inflammasome and autophagy. Front. Immunol. 11:591803. doi: 10.3389/fimmu.2020.591803, 33163006 PMC7583715

[ref7] Bodas M. Pehote G. Silverberg D. Gulbins E. Vij N. (2019). Autophagy augmentation alleviates cigarette smoke-induced CFTR-dysfunction, ceramide-accumulation and COPD-emphysema pathogenesis. Free Radic. Biol. Med. 131, 81–97. doi: 10.1016/j.freeradbiomed.2018.11.023, 30500419

[ref8] Bone N. B. Becker E. J. Husain M. Jiang S. Zmijewska A. A. Park D. W. . (2021). AMPK activates parkin independent autophagy and improves post sepsis immune defense against secondary bacterial lung infections. Sci. Rep. 11:12387. doi: 10.1038/s41598-021-90573-0, 34117280 PMC8196038

[ref9] Cavinato L. Genise E. Luly F. R. Di Domenico E. G. Del Porto P. Ascenzioni F. (2020). Escaping the phagocytic oxidative burst: the role of SODB in the survival of *Pseudomonas aeruginosa* within macrophages. Front. Microbiol. 11:326. doi: 10.3389/fmicb.2020.00326, 32210934 PMC7077434

[ref10] Chakraborty A. Bandyopadhaya A. Singh V. K. Kovacic F. Cha S. Oldham W. M. . (2024). The bacterial quorum sensing signal 2′-aminoacetophenone rewires immune cell bioenergetics through the Ppargc1a/Esrra axis to mediate tolerance to infection. eLife 13:RP97568. doi: 10.7554/eLife.97568, 39269443 PMC11398867

[ref11] Chakraborty A. Kabashi A. Wilk S. Rahme L. G. (2023). Quorum-sensing signaling molecule 2-aminoacetophenone mediates the persistence of *Pseudomonas aeruginosa* in macrophages by interference with autophagy through epigenetic regulation of lipid biosynthesis. MBio 14:e0015923. doi: 10.1128/mbio.00159-23, 37010415 PMC10127747

[ref12] Deng P. Uma Naresh N. Du Y. Lamech L. T. Yu J. Zhu L. J. . (2019). Mitochondrial UPR repression during *Pseudomonas aeruginosa* infection requires the bZIP protein ZIP-3. Proc. Natl. Acad. Sci. USA 116, 6146–6151. doi: 10.1073/pnas.1817259116, 30850535 PMC6442607

[ref13] Deng Q. Wang Y. Zhang Y. Li M. Li D. Huang X. . (2016). *Pseudomonas aeruginosa* triggers macrophage autophagy to escape intracellular killing by activation of the NLRP3 inflammasome. Infect. Immun. 84, 56–66. doi: 10.1128/IAI.00945-15, 26467446 PMC4694000

[ref14] Deretic V. (2021). Autophagy in inflammation, infection, and immunometabolism. Immunity 54, 437–453. doi: 10.1016/j.immuni.2021.01.018, 33691134 PMC8026106

[ref15] Deretic V. Levine B. (2018). *Autophagy* balances inflammation in innate immunity. Autophagy 14, 243–251. doi: 10.1080/15548627.2017.1402992, 29165043 PMC5902214

[ref16] Du Y. Guo H. Guo L. Miao J. Ren H. Liu K. . (2021). The regulatory effect of acetylation of HMGN2 and H3K27 on pyocyanin-induced autophagy in macrophages by affecting Ulk1 transcription. J. Cell. Mol. Med. 25, 7524–7537. doi: 10.1111/jcmm.16788, 34278675 PMC8335688

[ref17] Ferrari E. Monzani R. Villella V. R. Esposito S. Saluzzo F. Rossin F. . (2017). Cysteamine re-establishes the clearance of *Pseudomonas aeruginosa* by macrophages bearing the cystic fibrosis-relevant F508del-CFTR mutation. Cell Death Dis. 8:e2544. doi: 10.1038/cddis.2016.476, 28079883 PMC5386380

[ref18] Folkesson A. Jelsbak L. Yang L. Johansen H. K. Ciofu O. Høiby N. . (2012). Adaptation of *Pseudomonas aeruginosa* to the cystic fibrosis airway: an evolutionary perspective. Nat. Rev. Microbiol. 10, 841–851. doi: 10.1038/nrmicro2907, 23147702

[ref19] Galluzzi L. Baehrecke E. H. Ballabio A. Boya P. Bravo-San Pedro J. M. Cecconi F. . (2017). Molecular definitions of autophagy and related processes. EMBO J. 36, 1811–1836. doi: 10.15252/embj.201796697, 28596378 PMC5494474

[ref20] Ge J. Cao M. Zhang Y. Wu T. Liu J. Pu J. . (2025a). Inhibiting NLRP3 enhances cellular autophagy induced by outer membrane vesicles from *Pseudomonas aeruginosa*. Microbiol. Spectrum 13:e0181924. doi: 10.1128/spectrum.01819-24, 39873509 PMC11878092

[ref21] Ge J. Liu Y. Wu T. Yi S. Pu J. Gu J. . (2025b). Outer membrane vesicles from *Pseudomonas aeruginosa* induce autophagy-regulated pyroptosis in THP-1 cells. Arch. Microbiol. 207:54. doi: 10.1007/s00203-025-04264-9, 39928143

[ref22] Gomes L. C. Dikic I. (2014). Autophagy in antimicrobial immunity. Mol. Cell 54, 224–233. doi: 10.1016/j.molcel.2014.03.009, 24766886

[ref23] Gutierrez M. G. Master S. S. Singh S. B. Taylor G. A. Colombo M. I. Deretic V. (2004a). Autophagy is a defense mechanism inhibiting BCG and *Mycobacterium tuberculosis* survival in infected macrophages. Cell 119, 753–766. doi: 10.1016/j.cell.2004.11.038, 15607973

[ref24] Gutierrez M. G. Munafó D. B. Berón W. Colombo M. I. (2004b). Rab7 is required for the normal progression of the autophagic pathway in mammalian cells. J. Cell Sci. 117, 2687–2697. doi: 10.1242/jcs.01114, 15138286

[ref25] Hanada T. Noda N. N. Satomi Y. Ichimura Y. Fujioka Y. Takao T. . (2007). The Atg12-Atg5 conjugate has a novel E3-like activity for protein lipidation in autophagy. J. Biol. Chem. 282, 37298–37302. doi: 10.1074/jbc.C700195200, 17986448

[ref26] Hardy K. S. Tuckey A. N. Housley N. A. Andrews J. Patel M. Al-Mehdi A. B. . (2022). The *Pseudomonas aeruginosa* type III secretion system exoenzyme effector ExoU induces mitochondrial damage in a murine bone marrow-derived macrophage infection model. Infect. Immun. 90:e0047021. doi: 10.1128/IAI.00470-21, 35130452 PMC8929383

[ref27] Hauser A. R. (2009). The type III secretion system of *Pseudomonas aeruginosa*: infection by injection. Nat. Rev. Microbiol. 7, 654–665. doi: 10.1038/nrmicro2199, 19680249 PMC2766515

[ref28] Heimer S. R. Evans D. J. Stern M. E. Barbieri J. T. Yahr T. Fleiszig S. M. (2013). *Pseudomonas aeruginosa* utilizes the type III secreted toxin ExoS to avoid acidified compartments within epithelial cells. PLoS One 8:e73111. doi: 10.1371/journal.pone.0073111, 24058462 PMC3776860

[ref29] Hosokawa N. Hara T. Kaizuka T. Kishi C. Takamura A. Miura Y. . (2009). Nutrient-dependent mTORC1 association with the ULK1-Atg13-FIP200 complex required for autophagy. Mol. Biol. Cell 20, 1981–1991. doi: 10.1091/mbc.e08-12-1248, 19211835 PMC2663915

[ref30] Huang T. Pu Q. Zhou C. Lin P. Gao P. Zhang X. . (2020). MicroRNA-302/367 cluster impacts host antimicrobial defense via regulation of mitophagic response against *Pseudomonas aeruginosa* infection. Front. Immunol. 11:569173. doi: 10.3389/fimmu.2020.569173, 33117356 PMC7576609

[ref31] Jabir M. S. Hopkins L. Ritchie N. D. Ullah I. Bayes H. K. Li D. . (2015). Mitochondrial damage contributes to *Pseudomonas aeruginosa* activation of the inflammasome and is downregulated by autophagy. Autophagy 11, 166–182. doi: 10.4161/15548627.2014.981915, 25700738 PMC4502769

[ref32] Jabir M. S. Sulaiman G. M. Taqi Z. J. Li D. (2018). Iraqi propolis increases degradation of IL-1β and NLRC4 by autophagy following *Pseudomonas aeruginosa* infection. Microbes Infect. 20, 89–100. doi: 10.1016/j.micinf.2017.10.007, 29104144

[ref33] Jati S. Kundu S. Chakraborty A. Mahata S. K. Nizet V. Sen M. (2018). Wnt5A signaling promotes defense against bacterial pathogens by activating a host autophagy circuit. Front. Immunol. 9:679. doi: 10.3389/fimmu.2018.00679, 29686674 PMC5900007

[ref34] Jiang P. Nishimura T. Sakamaki Y. Itakura E. Hatta T. Natsume T. . (2014). The HOPS complex mediates autophagosome-lysosome fusion through interaction with syntaxin 17. Mol. Biol. Cell 25, 1327–1337. doi: 10.1091/mbc.E13-08-0447, 24554770 PMC3982997

[ref35] Jiang F. Wang X. Wang B. Chen L. Zhao Z. Waterfield N. R. . (2016). The *Pseudomonas aeruginosa* type VI secretion PGAP1-like effector induces host autophagy by activating endoplasmic reticulum stress. Cell Rep. 16, 1502–1509. doi: 10.1016/j.celrep.2016.07.012, 27477276

[ref36] Jin X. Zhang C. Lin S. Gao T. Qian H. Qu L. . (2024). Pec 1 of *Pseudomonas aeruginosa* inhibits bacterial clearance of host by blocking autophagy in macrophages. ACS Infect Dis 10, 2741–2754. doi: 10.1021/acsinfecdis.4c00096, 39047963

[ref37] Jorgensen I. Zhang Y. Krantz B. A. Miao E. A. (2016). Pyroptosis triggers pore-induced intracellular traps (PITs) that capture bacteria and lead to their clearance by efferocytosis. J. Exp. Med. 213, 2113–2128. doi: 10.1084/jem.20151613, 27573815 PMC5030797

[ref38] Junkins R. D. McCormick C. Lin T. J. (2014). The emerging potential of autophagy-based therapies in the treatment of cystic fibrosis lung infections. Autophagy 10, 538–547. doi: 10.4161/auto.27750, 24434788 PMC4077897

[ref39] Junkins R. D. Shen A. Rosen K. McCormick C. Lin T. J. (2013). Autophagy enhances bacterial clearance during *P. aeruginosa* lung infection. PLoS One 8:e72263. doi: 10.1371/journal.pone.0072263, 24015228 PMC3756076

[ref40] Kabeya Y. Mizushima N. Ueno T. Yamamoto A. Kirisako T. Noda T. . (2000). LC3, a mammalian homologue of yeast Apg8p, is localized in autophagosome membranes after processing. EMBO J. 19, 5720–5728. doi: 10.1093/emboj/19.21.5720, 11060023 PMC305793

[ref41] Kang D. Kirienko D. R. Webster P. Fisher A. L. Kirienko N. V. (2018). Pyoverdine, a siderophore from *Pseudomonas aeruginosa*, translocates into *C. elegans*, removes iron, and activates a distinct host response. Virulence 9, 804–817. doi: 10.1080/21505594.2018.1449508, 29532717 PMC5955448

[ref42] Kannan S. Audet A. Huang H. Chen L. J. Wu M. (2008). Cholesterol-rich membrane rafts and Lyn are involved in phagocytosis during *Pseudomonas aeruginosa* infection. J. Immunol. 180, 2396–2408. doi: 10.4049/jimmunol.180.4.2396, 18250449

[ref43] Kannan S. Audet A. Knittel J. Mullegama S. Gao G. F. Wu M. (2006). Src kinase Lyn is crucial for *Pseudomonas aeruginosa* internalization into lung cells. Eur. J. Immunol. 36, 1739–1752. doi: 10.1002/eji.200635973, 16791881

[ref44] Kaufmann S. H. E. Dorhoi A. Hotchkiss R. S. Bartenschlager R. (2018). Host-directed therapies for bacterial and viral infections. Nat. Rev. Drug Discov. 17, 35–56. doi: 10.1038/nrd.2017.162, 28935918 PMC7097079

[ref45] Kaushik S. Cuervo A. M. (2018). The coming of age of chaperone-mediated autophagy. Nat. Rev. Mol. Cell Biol. 19, 365–381. doi: 10.1038/s41580-018-0001-6, 29626215 PMC6399518

[ref46] Kim J. Kundu M. Viollet B. Guan K. L. (2011). AMPK and mTOR regulate autophagy through direct phosphorylation of Ulk1. Nat. Cell Biol. 13, 132–141. doi: 10.1038/ncb2152, 21258367 PMC3987946

[ref47] Kirienko N. V. Ausubel F. M. Ruvkun G. (2015). Mitophagy confers resistance to siderophore-mediated killing by *Pseudomonas aeruginosa*. Proc. Natl. Acad. Sci. USA 112, 1821–1826. doi: 10.1073/pnas.1424954112, 25624506 PMC4330731

[ref48] Kirienko N. V. Kirienko D. R. Larkins-Ford J. Wählby C. Ruvkun G. Ausubel F. M. (2013). *Pseudomonas aeruginosa* disrupts *Caenorhabditis elegans* iron homeostasis, causing a hypoxic response and death. Cell Host Microbe 13, 406–416. doi: 10.1016/j.chom.2013.03.003, 23601103 PMC3641844

[ref49] Klionsky D. J. Abdel-Aziz A. K. Abdelfatah S. Abdellatif M. Abdoli A. Abel S. . (2021). Guidelines for the use and interpretation of assays for monitoring autophagy (4th edition)1. Autophagy 17, 1–382. doi: 10.1080/15548627.2020.1797280, 33634751 PMC7996087

[ref50] Koyano F. Okatsu K. Kosako H. Tamura Y. Go E. Kimura M. . (2014). Ubiquitin is phosphorylated by PINK1 to activate parkin. Nature 510, 162–166. doi: 10.1038/nature13392, 24784582

[ref51] Kroken A. R. Gajenthra Kumar N. Yahr T. L. Smith B. E. Nieto V. Horneman H. . (2022). Exotoxin S secreted by internalized *Pseudomonas aeruginosa* delays lytic host cell death. PLoS Pathog. 18:e1010306. doi: 10.1371/journal.ppat.1010306, 35130333 PMC8853526

[ref52] Kushwaha A. Verma R. S. Agarwal V. (2022). *Pseudomonas aeruginosa* quorum-sensing molecule N-(3-oxododecanoyl) homoserine lactone induces calcium signaling-dependent crosstalk between autophagy and apoptosis in human macrophages. Cell. Signal. 99:110441. doi: 10.1016/j.cellsig.2022.110441, 35995303

[ref53] Lazarou M. Sliter D. A. Kane L. A. Sarraf S. A. Wang C. Burman J. L. . (2015). The ubiquitin kinase PINK1 recruits autophagy receptors to induce mitophagy. Nature 524, 309–314. doi: 10.1038/nature14893, 26266977 PMC5018156

[ref54] Levine B. Kroemer G. (2019). Biological functions of autophagy genes: a disease perspective. Cell 176, 11–42. doi: 10.1016/j.cell.2018.09.048, 30633901 PMC6347410

[ref55] Li Y. Gan C. P. Zhang S. Zhou X. K. Li X. F. Wei Y. Q. . (2014). FIP200 is involved in murine pseudomonas infection by regulating HMGB1 intracellular translocation. Cell. Physiol. Biochem. 33, 1733–1744. doi: 10.1159/000362954, 24923305 PMC4393741

[ref56] Li X. He S. Zhou X. Ye Y. Tan S. Zhang S. . (2016). Lyn delivers bacteria to lysosomes for eradication through TLR2-initiated autophagy related phagocytosis. PLoS Pathog. 12:e1005363. doi: 10.1371/journal.ppat.1005363, 26735693 PMC4703367

[ref57] Li R. Tan S. Yu M. Jundt M. C. Zhang S. Wu M. (2015). Annexin A2 regulates autophagy in *Pseudomonas aeruginosa* infection through the Akt1-mTOR-ULK1/2 signaling pathway. J. Immunol. 195, 3901–3911. doi: 10.4049/jimmunol.1500967, 26371245 PMC4592832

[ref58] Ligeon L. A. Moreau K. Barois N. Bongiovanni A. Lacorre D. A. Werkmeister E. . (2014). Role of VAMP3 and VAMP7 in the commitment of *Yersinia pseudotuberculosis* to LC3-associated pathways involving single- or double-membrane vacuoles. Autophagy 10, 1588–1602. doi: 10.4161/auto.29411, 25046114 PMC4206537

[ref59] Lim Y. Berry B. Viteri S. McCall M. Park E. C. Rongo C. . (2021). FNDC-1-mediated mitophagy and ATFS-1 coordinate to protect against hypoxia-reoxygenation. Autophagy 17, 3389–3401. doi: 10.1080/15548627.2021.1872885, 33416042 PMC8632273

[ref60] Lister P. D. Wolter D. J. Hanson N. D. (2009). Antibacterial-resistant *Pseudomonas aeruginosa*: clinical impact and complex regulation of chromosomally encoded resistance mechanisms. Clin. Microbiol. Rev. 22, 582–610. doi: 10.1128/CMR.00040-09, 19822890 PMC2772362

[ref61] Luciani A. Villella V. R. Esposito S. Brunetti-Pierri N. Medina D. Settembre C. . (2010). Defective CFTR induces aggresome formation and lung inflammation in cystic fibrosis through ROS-mediated autophagy inhibition. Nat. Cell Biol. 12, 863–875. doi: 10.1038/ncb2090, 20711182

[ref62] Martinez J. Malireddi R. K. Lu Q. Cunha L. D. Pelletier S. Gingras S. . (2015). Molecular characterization of LC3-associated phagocytosis reveals distinct roles for Rubicon, NOX2 and autophagy proteins. Nat. Cell Biol. 17, 893–906. doi: 10.1038/ncb3192 PMC4612372 26098576

[ref63] Maurice N. M. Bedi B. Sadikot R. T. (2018). *Pseudomonas aeruginosa* biofilms: host response and clinical implications in lung infections. Am. J. Respir. Cell Mol. Biol. 58, 428–439. doi: 10.1165/rcmb.2017-0321TR, 29372812 PMC5894500

[ref64] Miao E. A. Ernst R. K. Dors M. Mao D. P. Aderem A. (2008). *Pseudomonas aeruginosa* activates caspase 1 through Ipaf. Proc. Natl. Acad. Sci. USA 105, 2562–2567. doi: 10.1073/pnas.0712183105, 18256184 PMC2268176

[ref65] Mizushima N. Komatsu M. (2011). Autophagy: renovation of cells and tissues. Cell 147, 728–741. doi: 10.1016/j.cell.2011.10.026, 22078875

[ref66] Mizushima N. Kuma A. Kobayashi Y. Yamamoto A. Matsubae M. Takao T. . (2003). Mouse Apg16L, a novel WD-repeat protein, targets to the autophagic isolation membrane with the Apg12-Apg5 conjugate. J. Cell Sci. 116, 1679–1688. doi: 10.1242/jcs.00381, 12665549

[ref67] Mizushima N. Levine B. (2010). Autophagy in mammalian development and differentiation. Nat. Cell Biol. 12, 823–830. doi: 10.1038/ncb0910-823, 20811354 PMC3127249

[ref68] Mohankumar V. Ramalingam S. Chidambaranathan G. P. Prajna L. (2018). Autophagy induced by type III secretion system toxins enhances clearance of *Pseudomonas aeruginosa* from human corneal epithelial cells. Biochem. Biophys. Res. Commun. 503, 1510–1515. doi: 10.1016/j.bbrc.2018.07.071, 30031608

[ref69] Nakagawa I. Amano A. Mizushima N. Yamamoto A. Yamaguchi H. Kamimoto T. . (2004). Autophagy defends cells against invading group a Streptococcus. Science 306, 1037–1040. doi: 10.1126/science.1103966, 15528445

[ref70] Nakahira K. Haspel J. A. Rathinam V. A. Lee S. J. Dolinay T. Lam H. C. . (2011). Autophagy proteins regulate innate immune responses by inhibiting the release of mitochondrial DNA mediated by the NALP3 inflammasome. Nat. Immunol. 12, 222–230. doi: 10.1038/ni.1980, 21151103 PMC3079381

[ref71] Narendra D. Tanaka A. Suen D. F. Youle R. J. (2008). Parkin is recruited selectively to impaired mitochondria and promotes their autophagy. J. Cell Biol. 183, 795–803. doi: 10.1083/jcb.200809125, 19029340 PMC2592826

[ref72] Nie Q. Fang Z. Qin J. Wu M. Zhang Y. Ye L. . (2025). A unique amino acid, se-met, regulates autophagy and inflammation in *Pseudomonas aeruginosa* infection in mice through the PAK1/Akt1/mTOR and NF-κB signaling pathway. ACS Infect Dis 11, 1868–1879. doi: 10.1021/acsinfecdis.4c00987, 40472836

[ref73] Oku M. Sakai Y. (2018). Three distinct types of microautophagy based on membrane dynamics and molecular machineries. BioEssays 40:e1800008. doi: 10.1002/bies.201800008, 29708272

[ref74] Pang Z. Raudonis R. McCormick C. Cheng Z. (2019). Early growth response 1 deficiency protects the host against *Pseudomonas aeruginosa* lung infection. Infect. Immun. 88:e00678-19. doi: 10.1128/IAI.00678-19, 31611276 PMC6921661

[ref75] Pang Z. Xu Y. Zhu Q. (2021). Early growth response 1 suppresses macrophage phagocytosis by inhibiting NRF2 activation through upregulation of autophagy during *Pseudomonas aeruginosa* infection. Front. Cell. Infect. Microbiol. 11:773665. doi: 10.3389/fcimb.2021.773665, 35096638 PMC8790152

[ref76] Parkins M. D. Somayaji R. Waters V. J. (2018). Epidemiology, biology, and impact of clonal *Pseudomonas aeruginosa* infections in cystic fibrosis. Clin. Microbiol. Rev. 31, e00019–e00018. doi: 10.1128/CMR.00019-18, 30158299 PMC6148191

[ref77] Pavlik P. Velecka E. Spidlova P. (2024). Breaking the cellular defense: the role of autophagy evasion in Francisella virulence. Front. Cell. Infect. Microbiol. 14:1523597. doi: 10.3389/fcimb.2024.1523597, 39776438 PMC11703736

[ref78] Pu Q. Gan C. Li R. Li Y. Tan S. Li X. . (2017). Atg7 deficiency intensifies inflammasome activation and pyroptosis in Pseudomonas sepsis. J. Immunol. 198, 3205–3213. doi: 10.4049/jimmunol.1601196, 28258192 PMC5382979

[ref79] Ramírez-Estrada S. Borgatta B. Rello J. (2016). *Pseudomonas aeruginosa* ventilator-associated pneumonia management. Infect Drug Resist 9, 7–18. doi: 10.2147/IDR.S50669, 26855594 PMC4725638

[ref80] Rao L. De La Rosa I. Xu Y. Sha Y. Bhattacharya A. Holtzman M. J. . (2021). *Pseudomonas aeruginosa* survives in epithelia by ExoS-mediated inhibition of autophagy and mTOR. EMBO Rep. 22:e50613. doi: 10.15252/embr.202050613, 33345425 PMC7857434

[ref81] Rimessi A. Bezzerri V. Patergnani S. Marchi S. Cabrini G. Pinton P. (2015). Mitochondrial Ca2+−dependent NLRP3 activation exacerbates the *Pseudomonas aeruginosa*-driven inflammatory response in cystic fibrosis. Nat. Commun. 6:6201. doi: 10.1038/ncomms7201, 25648527

[ref82] Rodriguez Gonzalez C. Basílio-Queirós D. Neehus A. L. Merkert S. Tschritter D. Ünal S. . (2024). Human CFTR deficient iPSC-macrophages reveal impaired functional and transcriptomic response upon *Pseudomonas aeruginosa* infection. Front. Immunol. 15:1397886. doi: 10.3389/fimmu.2024.1397886, 39606224 PMC11601075

[ref83] Russell R. C. Tian Y. Yuan H. Park H. W. Chang Y. Y. Kim J. . (2013). ULK1 induces autophagy by phosphorylating Beclin-1 and activating VPS34 lipid kinase. Nat. Cell Biol. 15, 741–750. doi: 10.1038/ncb2757, 23685627 PMC3885611

[ref84] Ryu J. C. Kim M. J. Kwon Y. Oh J. H. Yoon S. S. Shin S. J. . (2017). Neutrophil pyroptosis mediates pathology of *P. aeruginosa* lung infection in the absence of the NADPH oxidase NOX2. Mucosal Immunol. 10, 757–774. doi: 10.1038/mi.2016.73, 27554297

[ref85] Sadikot R. T. Blackwell T. S. Christman J. W. Prince A. S. (2005). Pathogen-host interactions in *Pseudomonas aeruginosa* pneumonia. Am. J. Respir. Crit. Care Med. 171, 1209–1223. doi: 10.1164/rccm.200408-1044SO, 15695491 PMC2718459

[ref86] Saitoh T. Fujita N. Jang M. H. Uematsu S. Yang B. G. Satoh T. . (2008). Loss of the autophagy protein Atg16L1 enhances endotoxin-induced IL-1beta production. Nature 456, 264–268. doi: 10.1038/nature07383 18849965

[ref87] Sanjuan M. A. Dillon C. P. Tait S. W. Moshiach S. Dorsey F. Connell S. . (2007). Toll-like receptor signalling in macrophages links the autophagy pathway to phagocytosis. Nature 450, 1253–1257. doi: 10.1038/nature06421, 18097414

[ref88] Santoni K. Pericat D. Gorse L. Buyck J. Pinilla M. Prouvensier L. . (2022). Caspase-1-driven neutrophil pyroptosis and its role in host susceptibility to *Pseudomonas aeruginosa*. PLoS Pathog. 18:e1010305. doi: 10.1371/journal.ppat.1010305, 35849616 PMC9345480

[ref89] Sato H. Frank D. W. Hillard C. J. Feix J. B. Pankhaniya R. R. Moriyama K. . (2003). The mechanism of action of the *Pseudomonas aeruginosa*-encoded type III cytotoxin, ExoU. EMBO J. 22, 2959–2969. doi: 10.1093/emboj/cdg290, 12805211 PMC162142

[ref90] Smith E. E. Buckley D. G. Wu Z. Saenphimmachak C. Hoffman L. R. D'Argenio D. A. . (2006). Genetic adaptation by *Pseudomonas aeruginosa* to the airways of cystic fibrosis patients. Proc. Natl. Acad. Sci. USA 103, 8487–8492. doi: 10.1073/pnas.0602138103, 16687478 PMC1482519

[ref91] Tacconelli E. Carrara E. Savoldi A. Harbarth S. Mendelson M. Monnet D. L. . (2018). Discovery, research, and development of new antibiotics: the WHO priority list of antibiotic-resistant bacteria and tuberculosis. Lancet Infect. Dis. 18, 318–327. doi: 10.1016/S1473-3099(17)30753-3, 29276051

[ref92] Thurston T. L. Ryzhakov G. Bloor S. von Muhlinen N. Randow F. (2009). The TBK1 adaptor and autophagy receptor NDP52 restricts the proliferation of ubiquitin-coated bacteria. Nat. Immunol. 10, 1215–1221. doi: 10.1038/ni.1800, 19820708

[ref93] Thurston T. L. Wandel M. P. von Muhlinen N. Foeglein A. Randow F. (2012). Galectin 8 targets damaged vesicles for autophagy to defend cells against bacterial invasion. Nature 482, 414–418. doi: 10.1038/nature10744, 22246324 PMC3343631

[ref94] Tjahjono E. Kirienko N. V. (2017). A conserved mitochondrial surveillance pathway is required for defense against *Pseudomonas aeruginosa*. PLoS Genet. 13:e1006876. doi: 10.1371/journal.pgen.1006876, 28662060 PMC5510899

[ref95] Tjahjono E. McAnena A. P. Kirienko N. V. (2020). The evolutionarily conserved ESRE stress response network is activated by ROS and mitochondrial damage. BMC Biol. 18:74. doi: 10.1186/s12915-020-00812-5, 32600387 PMC7322875

[ref96] Wang J. Zhang L. Liu Y. Liu Y. Xiong A. Ran Q. . (2025). Epithelial Atg5 deficiency intensifies caspase-11 activation, fueling extracellular mtDNA release to activate cGAS-STING-NLRP3 axis in macrophages during *Pseudomonas* infection. MedComm 6:e70239. doi: 10.1002/mco2.70239, 40529614 PMC12167704

[ref97] Wei Y. Chiang W. C. Sumpter R. Mishra P. Levine B. (2017). Prohibitin 2 is an inner mitochondrial membrane mitophagy receptor. Cell 168, 224–238.e10. doi: 10.1016/j.cell.2016.11.042, 28017329 PMC5235968

[ref98] Wild P. Farhan H. McEwan D. G. Wagner S. Rogov V. V. Brady N. R. . (2011). Phosphorylation of the autophagy receptor optineurin restricts Salmonella growth. Science 333, 228–233. doi: 10.1126/science.1205405, 21617041 PMC3714538

[ref99] World Health Organization (2024). WHO Bacterial Priority Pathogens List, 2024: Bacterial Pathogens of Public Health Importance to Guide Research, Development and Strategies to Prevent and Control Antimicrobial Resistance. Geneva: World Health Organization.

[ref100] Wu Y. Chen M. Chen H. Pan L. Zhao J. Sun S. . (2025). CAPN1 promotes *Pseudomonas aeruginosa*-induced infection by interacting with TFEB and inhibiting autophagy. J. Innate Immun. 17, 176–197. doi: 10.1159/000543244, 40081346 PMC11906175

[ref101] Wu Y. Li D. Wang Y. Chen K. Yang K. Huang X. . (2016). *Pseudomonas aeruginosa* promotes autophagy to suppress macrophage-mediated bacterial eradication. Int. Immunopharmacol. 38, 214–222. doi: 10.1016/j.intimp.2016.04.044 27295610

[ref102] Wu Y. Li D. Wang Y. Liu X. Zhang Y. Qu W. . (2018). Beta-defensin 2 and 3 promote bacterial clearance of *Pseudomonas aeruginosa* by inhibiting macrophage autophagy through downregulation of early growth response Gene-1 and c-FOS. Front. Immunol. 9:211. doi: 10.3389/fimmu.2018.00211, 29487594 PMC5816924

[ref103] Xu J. Ma Y. Zhu X. Zhang J. Cheng Z. Wu W. . (2020). Enhanced autophagy promotes the clearance of *Pseudomonas aeruginosa* in diabetic rats with wounds. Ann Transl Med 8:1362. doi: 10.21037/atm-20-1019, 33313107 PMC7723545

[ref104] Yang C. S. Lee J. S. Rodgers M. Min C. K. Lee J. Y. Kim H. J. . (2012). Autophagy protein Rubicon mediates phagocytic NADPH oxidase activation in response to microbial infection or TLR stimulation. Cell Host Microbe 11, 264–276. doi: 10.1016/j.chom.2012.01.018, 22423966 PMC3616771

[ref105] Yang Z. S. Ma L. Q. Zhu K. Yan J. Y. Bian L. Zhang K. Q. . (2016). Pseudomonas toxin pyocyanin triggers autophagy: implications for pathoadaptive mutations. Autophagy 12, 1015–1028. doi: 10.1080/15548627.2016.1170256, 27159636 PMC4922443

[ref106] Yim W. W. Mizushima N. (2020). Lysosome biology in autophagy. Cell Discov. 6:6. doi: 10.1038/s41421-020-0141-7 PMC7010707 32047650

[ref107] Yuan K. Huang C. Fox J. Laturnus D. Carlson E. Zhang B. . (2012). Autophagy plays an essential role in the clearance of *Pseudomonas aeruginosa* by alveolar macrophages. J. Cell Sci. 125, 507–515. doi: 10.1242/jcs.094573, 22302984 PMC3283879

[ref108] Zhang Y. Chen X. L. Huang A. W. Liu S. L. Liu W. J. Zhang N. . (2016). Mortality attributable to carbapenem-resistant *Pseudomonas aeruginosa* bacteremia: a meta-analysis of cohort studies. Emerg Microbes Infect 5:e27. doi: 10.1038/emi.2016.22, 27004762 PMC4820673

[ref109] Zhang S. Stoll G. Pedro J. M. B. S. Sica V. Sauvat A. Obrist F. . (2018). Evaluation of autophagy inducers in epithelial cells carrying the ΔF508 mutation of the cystic fibrosis transmembrane conductance regulator CFTR. Cell Death Dis. 9:191. doi: 10.1038/s41419-017-0235-9, 29415993 PMC5833759

[ref110] Zhu P. Bu H. Tan S. Liu J. Yuan B. Dong G. . (2020). A novel cochlioquinone derivative, CoB1, regulates autophagy in *Pseudomonas aeruginosa* infection through the PAK1/Akt1/mTOR signaling pathway. J. Immunol. 205, 1293–1305. doi: 10.4049/jimmunol.1901346 32747503

